# Quantification of Hydroxylated Polybrominated Diphenyl Ethers (OH-BDEs), Triclosan, and Related Compounds in Freshwater and Coastal Systems

**DOI:** 10.1371/journal.pone.0138805

**Published:** 2015-10-14

**Authors:** Jill F. Kerrigan, Daniel R. Engstrom, Donald Yee, Charles Sueper, Paul R. Erickson, Matthew Grandbois, Kristopher McNeill, William A. Arnold

**Affiliations:** 1 Department of Civil, Environmental, and Geo- Engineering, University of Minnesota, Minneapolis, Minnesota, United States of America; 2 St. Croix Watershed Research Station, Science Museum of Minnesota, Marine on St. Croix, Minnesota, United States of America; 3 San Francisco Estuary Institute, Oakland, California, United States of America; 4 Pace Analytical Services Inc., Minneapolis, Minnesota, United States of America; 5 Institute for Biogeochemistry and Pollutant Dynamics, ETH Zurich, Zurich, Switzerland; 6 Department of Chemistry, University of Minnesota, Minneapolis, Minnesota, United States of America; NSYSU, TAIWAN

## Abstract

Hydroxylated polybrominated diphenyl ethers (OH-BDEs) are a new class of contaminants of emerging concern, but the relative roles of natural and anthropogenic sources remain uncertain. Polybrominated diphenyl ethers (PBDEs) are used as brominated flame retardants, and they are a potential source of OH-BDEs via oxidative transformations. OH-BDEs are also natural products in marine systems. In this study, OH-BDEs were measured in water and sediment of freshwater and coastal systems along with the anthropogenic wastewater-marker compound triclosan and its photoproduct dioxin, 2,8-dichlorodibenzo-*p*-dioxin. The 6-OH-BDE 47 congener and its brominated dioxin (1,3,7-tribromodibenzo-*p*-dioxin) photoproduct were the only OH-BDE and brominated dioxin detected in surface sediments from San Francisco Bay, the anthropogenically impacted coastal site, where levels increased along a north-south gradient. Triclosan, 6-OH-BDE 47, 6-OH-BDE 90, 6-OH-BDE 99, and (only once) 6’-OH-BDE 100 were detected in two sediment cores from San Francisco Bay. The occurrence of 6-OH-BDE 47 and 1,3,7-tribromodibenzo-*p*-dioxin sediments in Point Reyes National Seashore, a marine system with limited anthropogenic impact, was generally lower than in San Francisco Bay surface sediments. OH-BDEs were not detected in freshwater lakes. The spatial and temporal trends of triclosan, 2,8-dichlorodibenzo-*p*-dioxin, OH-BDEs, and brominated dioxins observed in this study suggest that the dominant source of OH-BDEs in these systems is likely natural production, but their occurrence may be enhanced in San Francisco Bay by anthropogenic activities.

## Introduction

Polybrominated diphenyl ethers (PBDEs) have been used as flame retardants in textiles, polyurethane foam furniture padding, and electronics since the 1970s. Mass produced to serve as non-covalently-bonded additives, PBDEs frequently enter the environment by leaching from products and are detected worldwide. Manufacturing facilities [1], sewage/wastewater effluent [1,2], and atmospheric deposition [3] are all known sources of PBDE pollution. San Francisco Bay is a global hotspot for PBDE contamination, likely a result of California’s early adoption of stringent flammability standards. In 2002, the San Francisco Regional Monitoring Program for Trace Substances (RMP) began monitoring PBDEs in water, surface sediments, and bivalves [4]. Since the ban of commercial mixtures in 2003 of Penta-BDE (which contains BDE-47, BDE-99, BDE-100, BDE-153, and BDE-154 –the most widespread and bioaccumulative congeners) and Octa-BDE, PBDE levels in the estuary have declined in fish, bivalves, bird eggs, and sediment [5]. The reservoir of previously released PBDEs and the debromination of deca-BDEs, however, are a continuing source of these less-substituted congeners of greatest concern.

Hydroxylated PBDEs (OH-BDEs) are abiotic and biotic transformation products of PBDEs [6–12], and they are also natural products in marine systems [13–17]. The position of the hydroxyl group (OH-) is potentially indicative of the source of OH-BDE congeners. OH-BDEs produced via oxidation of PBDEs may have the OH- in the *ortho-*, *meta-*, or *para-* position relative to the ether bridge, whereas the metabolically produced OH-BDEs have the OH- primarily in the *ortho*-position [6,9]. Studies have shown OH-BDE formation via metabolic oxidation of PBDEs in rats [7], PBDE oxidation in the atmosphere by OH radicals [8], photochemical formation from brominated phenols [11], potentially during oxidation stages in wastewater and sewage treatment [10,18], and recently photochemically from PBDEs in aqueous solutions [12]. Recent evidence suggests that the natural production of OH-BDEs occurs by the coupling of simple bromophenols by both marine bacteria [14] and an enzyme isolated from red algae [19]. Although strong genetic evidence is lacking, studies suggest that red algae and cyanobacteria associated with marine sponges are potential OH-BDE producers independently and/or through associations with bacteria [13,14,16,17,20–22].

OH-BDEs have been detected in higher trophic levels, such as Baltic salmon [23], polar bears [24], bald eagles [25], and human plasma [26]. The highest reported level was 150 ng/g dry weight (dw) in red algae from the Baltic Sea [13]. In marine sediments, the mean concentration of 6-OH-BDE 47 was 22 ± 2.3 pg/g dw in Liaodong Bay, China [27] and levels ranged from 11.4 to 128 pg/g dw in the East China Sea [28]. In fresh waters, observed OH-BDEs levels ranged from 34 − 390 pg/L in South Korean rivers [29] and 2.2–70 pg/L in Lake Ontario and the Detroit River [8]. A recent study reported ΣOH-BDEs fluxes of 15 to 170 pg/m^2^/day in rain and 3.5 to 190 pg/m^2^/day in snow [8]. Furthermore, tetra- (6-OH-BDE 28 and 47) and penta-brominated (6-OH-BDE 90 and 99) OH-BDEs have been detected in wastewater effluents, generally at 1–10 ng/L levels [10,18].

OH-BDEs are either equivalent or more potent endocrine disruptors and neurotoxins than the precursor PBDEs [30,31]. Studies investigating the toxic effects of OH-BDEs have reported uncoupling of oxidative phosphorylation in zebrafish [32,33], indirect estrogenic effects in rats [34], disruption of thyroid function and neurological development via prenatal exposure in humans [35], and effects on hormone transport in gulls [36]. Also, OH-BDE congeners can form polybrominated dibenzo-*p*-dioxins (PBDDs) as photoproducts in natural waters [37,38]. The phototransformation occurs only in OH-BDE congeners with a bromine *ortho* to ether linkage and an *ortho* OH- on the adjacent phenyl ring. PBDDs also have anthropogenic sources such as formation by incineration of brominated flame retardants [39–43], and they too are also natural products in marine environments [13,44–47]. Studies have shown PBDDs have the same or greater toxicity than their chlorinated analogues, polychlorinated dibenzo-*p*-dioxins (PCDDs) [48–51].

Triclosan (5-chloro-2-(2,4-dichlorophenoxy)phenol) is an antibacterial agent in various consumer products, best known for its use in hand soaps and toothpaste. Triclosan is chemically similar to OH-TriBDE, except that triclosan is chlorinated, not brominated, and forms 2,8-dichlorodibenzo-p-dioxin (2,8-DiCDD) via photolysis in aquatic systems [52,53]. Triclosan was first produced in the 1960s [54], and the vast majority of triclosan-containing products are washed down the drain. Triclosan removal efficiencies in wastewater treatment plants (WWTPs) are >90% with conventional activated sludge treatment [55]. Even with high removal efficiencies, triclosan is frequently detected in wastewater effluents [56–59], which is the primary source of this pollutant in surface waters [60] and sediments [61–63] downstream from WWTPs. Negligible loadings come via run-off from wastewater sludge applied to agricultural fields [60]. A 30-state survey of wastewater-impacted streams and rivers detected triclosan in 57.6% of the sampled locations and reported a median and maximum concentration of 140 ng/L and 2.3 μg/L, respectively [64]. Triclosan accumulation rates in eight Minnesota lakes mirrored increased usage in consumer products, and overall levels were a function of the magnitude of wastewater input relative to lake area [61]. Triclosan may inhibit growth of various coastal microalgae and cyanobacteria and has toxic effects on freshwater and marine invertebrates and fish [65–68].

The objective of this research was to ascertain the importance of biosynthetic and anthropogenic OH-BDEs as brominated dioxin sources using the close structural analogue, triclosan, as an anthropogenic marker compound to assess the role of wastewater as a potential source. PBDEs may have large inputs from wastewater effluent, industry, the atmosphere, and other sources, whereas wastewater effluent is the primary source of triclosan. Because the onset of production and use of triclosan and PBDEs followed a similar timeline, we hypothesized that co-occurrence of triclosan and PBDEs/OH-BDEs could indicate a common anthropogenic source for the compounds. In this study we 1) measured OH-BDE congeners and triclosan in sediment and surface waters and 2) measured the levels of OH-BDE-derived brominated dioxins in surface sediments and correlated them with triclosan, triclosan-derived dioxin, and PBDEs levels/trends. Sediments from WWTP-impacted freshwater lakes (Lake Pepin, Lake St. Croix, and East Gemini Lake, MN), a relatively pristine marine environment (Point Reyes National Seashore, CA), and a WWTP-impacted estuary (San Francisco Bay, CA) were collected for this study. The OH-BDEs investigated in this study were selected because they: (1) were all capable of forming dioxins via photolysis, and (2) had different sources (anthropogenic and/or natural). Of the target OH-BDE congeners investigated, some have known natural and anthropogenic origins (6-OH-BDE 47, 6-OH-BDE 90, and 6-OH-BDE 99), whereas others are not known to be natural products (6’-OH-BDE 100 and 6’-OH-BDE 118). Only three brominated dioxins were included in this study due to commercial availability limitations. The photoproducts 1,3,7-TriBDD, 1,2,4,8-TeBDD, and 2,3,7,8-TeBDD (the most toxic PBDD) of 6-OH-BDE 47, 6-OH-BDE 99, and 6’-OH-BDE 118, respectively, were measured. The OH-BDE levels were compared with PBDE, PBDD, triclosan, and 2,8-DiCDD levels/trends.

## Materials and Methods

San Francisco Bay surface waters were collected by the San Francisco Estuary Institute (SFEI) and Applied Marine Sciences during a regularly scheduled RMP water sampling cruise aboard the vessel *RV Turning Tide*. A water sample was collected from RMP station LSB055W (GPS coord: 37.48458, -122.11815) on July 31, 2013, and from station BG30 (38.02041, -121.80537) on August 8, 2013. Water samples were collected into cleaned amber glass 4-L jugs, and stored on wet ice (~4°C) in a dark cooler while on board the vessel. Samples were shipped on liquid ice packs to the University of Minnesota where they were filtered with pre-combusted glass fiber filters, acidified to pH 3, and stored at 4°C.

**Fig 1:**
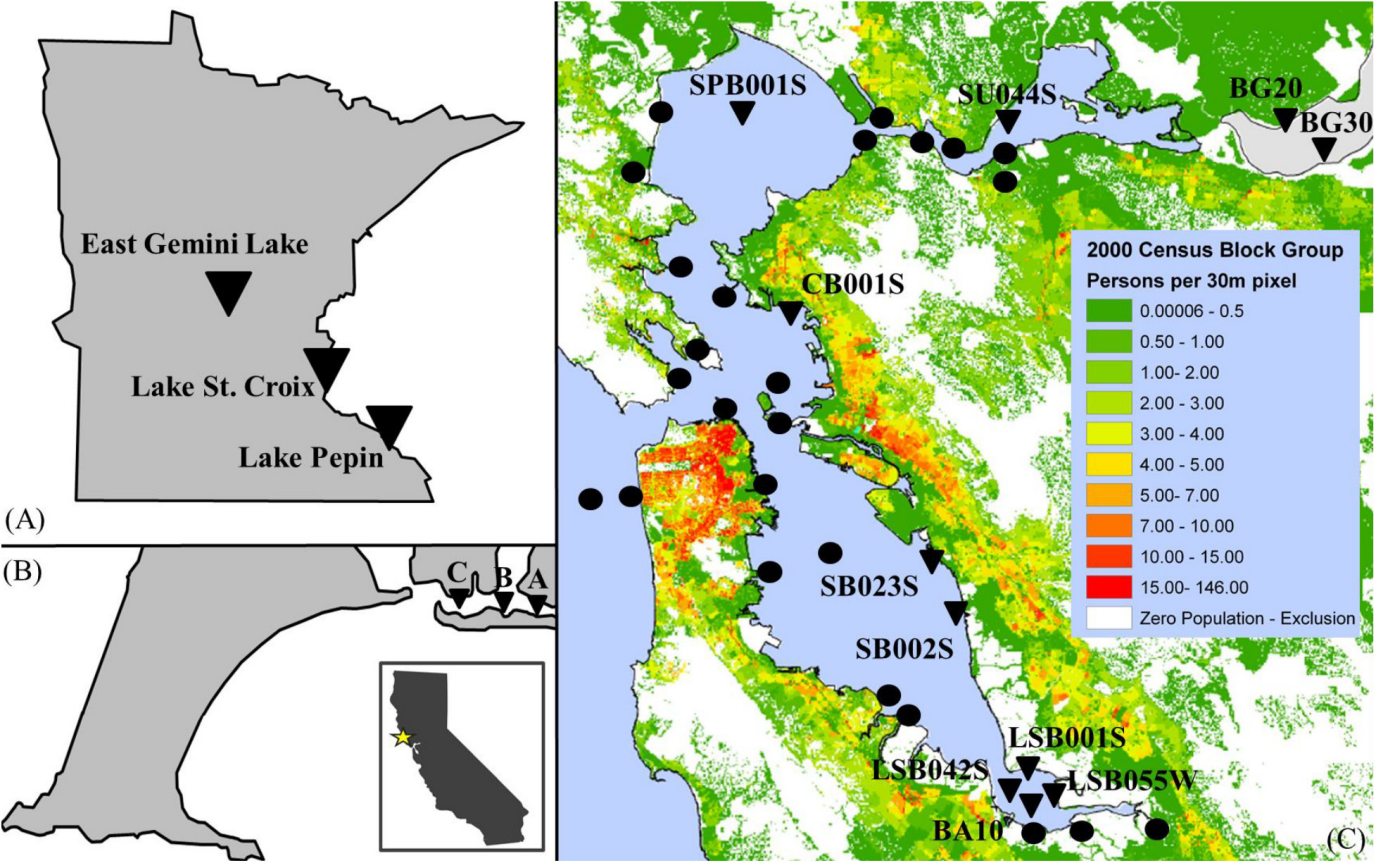
Maps of Minnesota (A) and California (B and C) sampling locations. (A) East Gemini Lake, Lake St. Croix, and Lake Pepin in Minnesota; (B) Point Reyes National Seashore, CA, and (C) 2000 census population density for the San Francisco Bay region generated by Dasymetric (ArcGIS10x) software courtesy of the U.S. Geological Survey with wastewater outfalls (black circle) and surface sediments, cores, and surface waters collection sites (black triangles).

San Francisco Bay surface sediments were collected between August 22 and August 31, 2011 on the *RV Endeavor* at locations shown in Fig 1 (GPS coordinates located below). The sampling scheme was designed as a spatially distributed unbiased representative sampling of the habitat resource. Surface sediments were collected using a Van Veen grab, with a composite of the top 5 cm of sediment from each site. Sediment cores of 50–60 cm in length were collected from RMP sites in Central Bay (Station CB001S, GPS 37.87645, -122.36132) and South Bay (Station SB002S, GPS 37.61025, -122.16757). The sediment cores were collected using a piston corer equipped with a 70-cm polycarbonate core barrel and operated from the water surface by Mg-alloy drive rods. Cores were extruded while on board the vessel and sectioned at 2- or 4-cm intervals. Push-cores were collected at low tide in shallow waters of the Limantour Estero at three sites (A: GPS 38.031225, -122.903838; B: GPS 38.031725, -122.90855; C: GPS 38.032036, -122.91358) at Point Reyes National Seashore on August 20, 2011 and extruded at 5 or 6-cm intervals. All sediment samples were placed into glass sample jars with foil-lined lids, frozen in the field on dry ice, and transported to the University of Minnesota. The cores from Lake Pepin, East Lake Gemini and Lake St. Croix were previously collected in 2010 (July–September) by Anger et al. [61] using a piston corer as described above.

**Fig 2:**
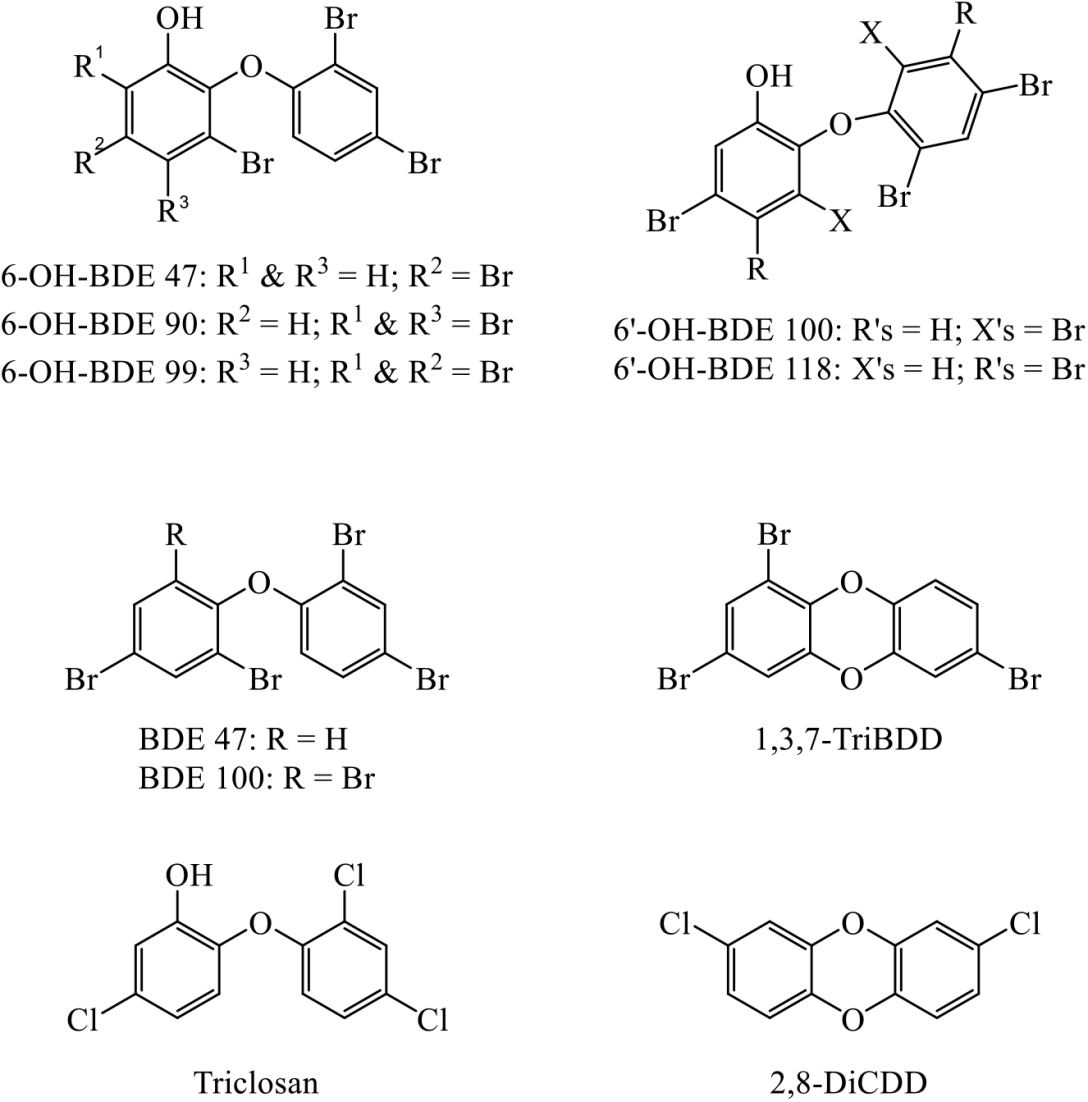
Chemical structures of OH-BDEs, PBDEs, triclosan, and polyhalogenated dibenzo-*p*-dioxins (PXDDs).

The structures of the target analytes are shown in Fig 2. 6-OH-BDE 47, 6-OH-BDE 99, 6’-OH-BDE 100, and 6’-OH-BDE 118 were synthesized and purified as described previously [37,69]. The synthesis of 6-OH-BDE 90 was performed according to Hensley et al. [18]. Note that the impurity of 6’-OH-BDE 100 was most likely due to a structural rearrangement [37]. Triclosan (TCS, >97%) was purchased from Sigma Aldrich. The ^13^C_12_-triclosan (^13^C_12_-TCS) (50 μg/mL in methanol, >99%), ^13^C_12_-6-OH-BDE 47 (50 μg/mL in methanol, >99%), and ^13^C_12_-6’-OH-BDE 100 (50 μg/mL in toluene, >99%), were purchased from Wellington Laboratories. The dioxins 1,3,7-TriBDD (10 μg/mL in toluene), 1,2,4,7/1,2,4,8-TeBDD-mixed (10 μg/mL in toluene), 2,3,7,8-TeBDD (1 mg), and 2,8-DiCDD (50 μg/mL in isooctane) and were purchased from AccuStandard, as well as the brominated and chlorinated surrogates ^13^C_12_-2,3,7,8-TeBDD (99%, 5 μg/mL in nonane) and ^13^C_12_-2,3-DiCDD (99%; 50 μg/mL), respectively. Sand (S25516A) and sulfuric acid were from Fisher Scientific. Ammonium acetate was from Mallinckrodt. Ultrapure water (18.2 MΩ-cm) was generated using a Millipore Simplicity UV purification system. All organic solvents used were HPLC grade, expect for methyl-*tert*-butyl ether (MTBE) which was ACS grade (>99%). Ultra-high purity and industrial-grade nitrogen were purchased from Matheson.

### Radiometric Dating

The Central and South Bay cores from San Francisco Bay were dated by ^210^Pb using isotope-dilution, alpha spectrometry methods and the constant flux:constant sedimentation (cf:cs) model [70,71]. The Central Bay core was also analyzed for ^137^Cs by gamma spectrometry to provide a supplemental dating marker to validate the ^210^Pb chronology. The Lake Pepin core was dated by stratigraphic correlation of whole-core magnetic susceptibility profiles with a radiometrically-dated master core collected previously from the same location [72].

### Surface Water and Sediment Extraction Methods

The solid phase extraction method and silica column clean-up for isolating OH-BDEs in surface waters was adapted from Buth et al [62] and a detailed explanation can be found in S1 Appendix. Subsamples of sediments were analyzed for moisture content and loss-on-ignition. Samples were weighed after being heated for 12 hours at 105°C, 4 hours at 550°C, and 2 hours at 1000°C to determine water, organic, and carbonate content, respectively. Sediments (~12 g dw) were freeze dried for 3–5 days and stored at -20°C until extraction. The accelerated solvent extraction (ASE) method for OH-BDEs in sediments was adapted from Anger et al [61], and a detailed explanation can be found in S1 Appendix.

Between 1 and 20 g (dw) of each sediment sample were extracted separately from OH-BDE analysis to be analyzed for 2,8-DiCDD and the targeted PBDD congeners. For all cores, samples were spiked with nineteen ^13^C_12_-labeled di- through octa-CDD/F isomers and with ^13^C_12_-labeled 2,3,7,8-TeBDD as isotope dilution surrogates. The core samples were then analyzed using an expanded version of U.S. EPA Method 1613B [73]. The extraction and high-resolution gas chromatography-high-resolution mass spectrometry (HRGC-HRMS) analysis are described in S1 Appendix.

### LC-MS/MS Method

Extracts were analyzed with a Waters nanoAcquity capillary high performance liquid chromatograph (LC) equipped with a Thermo Scientific TSQ Ultra AM MS-Q^3^ tandem mass spectrometer (MS/MS) using a negative electrospray ionization (ESI) source. The analytical method was adapted from Feo et al [74]. The stationary phase was a Thermo Hypersil Gold column (150 × 0.5 mm, 3μm) heated at a constant 30°C. The injection volume was 8 μL. The mobile phase was a binary gradient with (A) 3:2 15 mM ammonium acetate:MeOH and (B) acetonitrile with a flowrate of 15 μL/min. An initial 25% B ramped up to 40% B by 5 minutes, 46% B by 10 min, 48% B by 23 min, and 80% B at 25 min. Until 27 min, B remained at 80% and then ramped down to 25% B for a 10 min re-equilibration. A single reaction monitoring (SRM) transition was used for chemical quantification, in addition to another SRM transition to confirm the identity of the chemical (S1 Table). Instrument blanks (50:50 H_2_O:acetonitrile) were run every 7 or 8 samples to evaluate contamination via sample injections.

The mass spectrometer was infused with ^13^C_12_-TCS (30 mg/L in 50:50 H_2_O:acetonitrile) at the beginning of each analysis to optimize MS/MS parameters which varied slightly between runs due to the high sensitivity of the instrument. Typical optimized values were: collision energy: 11; scan time: 0.15 s; Q_1_/Q_3_: 0.7; spray voltage: 2700 V; sheath gas pressure: 11 psi; capillary temperature: 300°C; and collision pressure: 0.9 mTorr. Also, it was necessary to run a sediment extract two or three times at the beginning of each sequence to acquire consistent analyte signals.

Additional experimental and analytical details including cleaning protocols and calculation of absolute and relative recoveries and analyte concentrations using response factors are in S2 Appendix.

## Results

### Analytical Method Performance

The LC-MS/MS method for triclosan and OH-BDEs quantification separated the analytes of interest. Typical chromatograms for standards and samples can be seen in S1 Fig. It was determined that 6’-OH-BDE 100, labeled and unlabeled, transformed into another unknown OH-PentaBDE (retention time of 15.30 min in S1 Fig). This transformation was enhanced during the sediment extraction (using accelerated solvent extraction) and the transformation peak was slightly less retained than the (^13^C_12_-)6’-OH-BDE 100 during LC-MS/MS analysis. The sum of these two peak areas ((^13^C_12_-)6’-OH-BDE 100 and its transformation product) was used to account for the total presence of (^13^C_12_-)6’-OH-BDE 100. It should be noted that matrix effects in the samples caused shifts in retention times among samples, thus whenever possible internal standards were used to corroborate a peak’s identity. Not all analytes, however, had commercially available isotope labeled congeners. The difference of retention times between internal standard and analyte, therefore, was used to confirm the peak’s identity when no internal standard was available. Furthermore, other studies quantified penta- and tetrabrominated OH-BDEs that were not included in this study (e.g. 2’-OH-BDE 68). It is possible that an unknown OH-BDE co-eluted in environmental samples, but it unlikely considering the separation achieved in the work from which our method was derived [74].

Linear calibration curves ranged from 2–500 μg/L for OH-BDEs and 1–400 μg/L for triclosan and were of high quality (R^2^ > 0.98). The limits of detection (LOD) and quantification (LOQ) were calculated from the method blanks. The area in the blanks at the same retention times as the analytes was integrated and multiplied by 3 or 10 for the LOD and LOQ, respectively. Because 6’-OH-BDE 100 was detected in a single sample and 6’-OH-BDE 118 in no samples, the lowest concentration of the calibration curve was used to calculate an alternative LOQ for these two chemicals. Due to the variability of instrument’s sensitivity, LOQs ranged from 16–27 pg/g and 0.04–0.07 ng/L for triclosan in sediment and water, respectively, and 2–28 pg/g and 0.004–0.17 ng/L for OH-BDEs in sediment and water, respectively (Table 1). LODs ranged from 5–8 pg/g and 0.01–0.02 ng/L for triclosan in sediment and water, respectively, and 0.6–6.4 pg/g and 0.001–0.05 ng/L for OH-BDEs in sediment and water, respectively (see Table 1).

**Table 1:** Limits of detection and quantification for triclosan and OH-BDEs in water (ng/L) and sediment (pg/g) samples.

	LODs		LOQs	
*Chemicals*	*Water (ng/L)*	*Sediment (pg/g)*	*Water (ng/L)*	*Sediment (pg/g)*
Triclosan	0.01–0.02	5–8	0.04–0.07	16–27
6-OH-BDE 47	0.03–0.05	0.6–2.4	0.10–0. 17	2–8
6-OH-BDE 90	0.002–0.005	0.9–6.4	0.007–0.015	3–21
6’-OH-BDE 99	0.001–0.014	0.6–5.4	0.004–0.046	2–18
6’-OH-BDE 100*	N/A	N/A	0.1	28
6’-OH-BDE 118*	N/A	N/A	0.08	8

*Not detected in sample, LOQ was determined by lowest concentration of the calibration curve

The sediment and water concentrations above LOQ were calculated using isotope dilution analysis and were recovery corrected. The absolute recoveries were calculated for the isotope labeled compound (Table 2), and details are located in S2 Appendix. The relative recoveries for triclosan and OH-BDEs ranged from 44–133% in sediment and 70–134% in water samples, respectively (see Table 3). Note that lower recoveries increase the uncertainty in reported concentrations, but should not alter observed trends for each analyte. See S2 Table for the absolute and relative recoveries for ^13^C_12_-PXDDs and PXDDs, respectively. The dry density and percent organic, carbonate, and inorganic for every core interval and surface sediment was determined, and results are located in S3 Table and S2 Fig.

**Table 2:** Absolute recovery (%) of isotope labeled compounds in sediment and water matrices in *n* number of samples.

Site	^13^C_12_-TCS	^13^C_12_-6-OH-BDE 47	^13^C_12_-6’-OH-BDE 100	n
Surface Water	74 ± 24	54 ± 19	43 ± 13	17
South Bay Core	50 ± 21	42 ± 21	41 ± 16	17
Central Bay Core	78 ± 18	45 ± 11	36 ± 13	17
Surface Sediments	24 ± 12	21 ± 8	11 ± 4	10
Point Reyes National Seashore	71 ± 51	62 ± 31	41 ± 24	13

**Table 3:** Relative recovery (%) of analytes in sediment and water.

Chemical	Sediment	Water
Triclosan	133 ± 52	134 ± 12
6-OH-BDE 47	99 ± 8	104 ± 5
6-OH-BDE 90	72 ± 27	100 ± 24
6-OH-BDE 99	82 ± 33	93 ± 20
6’-OH-BDE 100	55 ± 9	117 ± 12
6’-OH-BDE 118	44 ± 21	70 ± 10

### Contaminant Levels in Surface Water and Surface Sediment Samples

6-OH-BDE 90 levels were elevated in the southern surface waters (LSB055W, 40 pg/L) relative to the northern surface water (BG30, < 12 pg/L), see Table 4. The other naturally produced OH-BDEs, 6-OH-BDE 47 and 6-OH-BDE 99, were not detected in the BG30 sample, but were detected (< 129 pg/L and < 19 pg/L, respectively) in the LSB055W sample. The anthropogenic OH-BDEs, 6’-OH-BDE 100 and 6’-OH-BDE 118, were not detected in any water sample. Triclosan concentrations were elevated in LSB055W (68 ± 26 ng/L) compared to the outlet of the San Joaquin River (BG30, 17 ± 9 ng/L). The salinity near the Sacramento and San Joaquin River outlets was low, 0.1 and 0.2 psu respectively, due to the freshwater input of the rivers. The salinity was fairly uniform (25.2 ± 1.9 psu) in the Central, South, and Lower South bays. Salinity measurements were taken a month after the sediments were collected, and most salinity values in Table 5 were taken at nearby collection points (see S4 Table for GPS coordinates).

**Table 4:** Concentrations (ng/L) of triclosan and OH-BDEs in surface waters.

Surface Water Levels (ng/L)		
Chemical	BG30	LSB055W
Triclosan	17 ± 9	68 ± 26
6-OH-BDE 47	ND	< 0.129
6-OH-BDE 90	< 0.012 ^*a*^	0.040 ^*b*^
6-OH-BDE 99	ND	< 0.019 ^*a*^

^*a*^ One replicate > LOD and < LOQ, with other replicates < LOD.

^*b*^ One replicate >LOQ, two replicates >LOD and <LOQ, and one replicate <LOD.

ND denotes concentration < LOD.

**Table 5:** Concentrations of triclosan, PBDEs, 6-OH-BDE 47, and PXDDs in San Francisco Bay sediments and salinity in surface waters.

Site Name (units)	Sample ID^a^	Latitude	Longitude	Triclosan (ng/g)	2,8-DiCDD (pg/g)	BDE 47^b^ (pg/g)	BDE 100^b^ (pg/g)	6-OH-BDE 47^c^ (pg/g)	1,3,7-TriBDD^d^ (pg/g)	Salinity^b^ (psu)
Sacramento River	BG 20	38.0583	-121.81407	0.17	12	46	12	ND	ND	0.1
Sacramento River	BG 30	38.02285	-121.80845	0.11	8	ND	ND	ND	1.5	0.2
Suisun Bay	SU044S	38.07597	-122.05687	0.21	15	30	ND	< 8.1^e^	3	5.4^f^
San Pablo Bay	SPB001S	38.07262	-122.38622	2.03	38	318	57	< 8.1^e^	1.8	18.3^f^
Central Bay	CB001S	37.87645	-122.36132	4.32	58	513	93	23.0	ND	28.4^f^
South Bay	SB023S	38.10478	-122.39208	2.45	110	137	23	82.5	11	26.6^f^
South Bay	SB002S	38.01615	-122.34122	2.30	150	226	40	263.8	15	24.3^f^
Lower South Bay	LSB001S	37.49168	-122.09868	6.00	N/A	590	105	16.5	N/A	24^f^
Lower South Bay	LSB042S	37.47168	-122.09555	5.47	160	273	35	188.2	7.2	23.9^f^
Coyote River	BA10	37.46812	-122.06385	4.64	120	524	106	12.8	6.4	24.1^f^

^a^ Sample IDs are those used by SFEI for these sampling locations in their Regional Monitoring Program

^b^ San Francisco Estuary Institute [75]

^c^ OH-BDEs with concentrations < LOD are not shown, includes: 6-OH-BDE 90, 6-OH-BDE 99, 6’-OH-BDE 100, and 6’-OH-BDE 118

^d^ PBDDs with concentrations < LOD are not shown, includes: 1,2,4,7/1,2,4,8-TeBDD, and 2,3,7,8-TeBDD

^e^ Concentration > LOD and < LOQ

^f^ Measured at nearby sites, see S4 Table.

ND denotes concentration < LOD

N/A denotes a sample that was not analyzed for a specific compound.

6-OH-BDE 47 and 1,3,7-TriBDD were the only OH-BDE and brominated dioxin, respectively, detected in San Francisco Bay surface sediments. Sediments near the northern rivers outlets had low to non-detected levels of 6-OH-BDE 47 and 1,3,7-TriBDD. Concentrations of 6-OH-BDE 47 (< 8.1–263.8 pg/g) and 1,3,7-TriBDD (3–15 pg/g) varied throughout the rest of the estuary with higher levels in the South and Lower South Bay (see Table 5). 6-OH-BDE 47 levels were higher than 1,3,7-TriBDD (2–36×) in San Francisco Bay surface sediments.

The relevant precursor PBDEs of anthropogenic 6-OH-BDE 47 are BDE 47 and BDE 100 (∑PBDE(47 +100)). The major formation pathways of 6-OH-BDE 47 is addition of–OH to the ring (BDE 47) and replacement of a–Br by–OH (BDE 100). The SFEI monitors approximately 50 PBDEs congeners in San Francisco Bay sediments and the entire data set is available at http://www.sfei.org/rmp/wqt [75]. The levels of BDE 47 and 100 shown in Table 5 originated from this data set. There were low to negligible levels of triclosan, BDE 47, and BDE 100 in surface sediments near the Sacramento and San Joaquin rivers in the northern part of the estuary, but higher and relatively uniform concentrations (2–6 ng/g for triclosan, 137–590 pg/g for BDE 47, and 23–106 pg/g for BDE 100) across the Central, South, and Lower South bays (see Table 5). A significant and positive correlation was seen between ∑PBDE(47 +100) and triclosan (p = 0.001, R^2^ = 0.75; S3 Fig). There was no significant correlation between 6-OH-BDE 47 and either ∑PBDE(47 +100) (p = 0.89, R^2^ = 0.002) or triclosan (p = 0.50, R^2^ = 0.057) in the surface sediments (S3 Fig). Furthermore, both 6-OH-BDE 47 and triclosan had positive and significant correlations with their respective photochemically produced dioxins (S3 Fig).

Previous work by Anger et al [61] and Buth et al [62] showed increasing levels of triclosan and 2,8-DiCDD in Minnesota lake sediments, including Lake Pepin, East Lake Gemini, and Lake St. Croix, since the mid-1960s. The same Lake Pepin samples analyzed by Anger et al [61] were re-analyzed using this study’s LC-MS/MS method, and no OH-BDEs were detected. Yet, 1,3,7-TriBDD was detected in three Lake Pepin sediments in core intervals dated to 2009, 2005, and 1997 at 2, 2.1, and 1.2 pg/g, respectively. No brominated dioxins were detected in core sediments dated to 1992–1944 in Lake Pepin, nor in any sediments from Lake St Croix and East Lake Gemini. Thus, wastewater effluent is a known source of anthropogenic chemicals in these freshwater lakes, yet no OH-BDEs, which must arise from anthropogenic sources in these freshwaters, were detected in these sediments.

At the relatively pristine marine site (Point Reyes National Seashore), 6-OH-BDE 47 and 1,3,7-TriBDD concentrations ranged from non-detected to 36.3 pg/g and non-detected to 2.4 pg/g, respectively. No other OH-BDEs or brominated dioxins were detected in these marine sediments. Low levels of the anthropogenic marker triclosan (0.02–0.55 ng/g) and 2,8-DiCDD (7–10 pg/g) were detected in these cores (Table 6), suggesting that the measured OH-BDEs at Point Reyes National Seashore originate from biological production and not from anthropogenic PBDEs.

**Table 6:** Concentration of triclosan, 2,8-DiCDD, 6-OH-BDE 47, and 1,3,7-TriBDD in three sediment cores (A, B, & C) at Point Reyes National Seashore.

Depth (cm)	Triclosan (ng/g)			2,8-DiCDD (pg/g)			6-OH-BDE 47^c^ (pg/g)			1,3,7-TriBDD^d^ (pg/g)		
* *	*A*	*B*	*C*	*A*	*B*	*C*	*A*	*B*	*C*	*A*	*B*	*C*
0–5	0.12	0.21	0.55	7.5	5.4	7.1	<8.1^b^	<8.1	14.2	2.4	0.99	1.2
5–10	0.02	0.19	0.23	8.2	9.1	7.3	9.4	ND	21.9	2.4	ND	1.7
10–15^a^	0.17	0.20	0.31	10	7	8.8	<8.1	<8.1	36.3	3	ND	1.8

ND denotes analyte levels below LOD

^a^ Final depth for core ‘C’ is 16 cm

^b^ Concentration > LOD and < LOQ

^c^ OH-BDEs with concentrations < LOD are not shown, includes: 6-OH-BDE 90, 6-OH-BDE 99, 6’-OH-BDE 100, and 6’-OH-BDE 118

^d^ PBDDs with concentrations < LOD are not shown, includes: 1,2,4,7/1,2,4,8-TeBDD, and 2,3,7,8-TeBDD

### Temporal Trends for 6-OH-BDE 47 and Triclosan

Lead-210 activities were low throughout both San Francisco Bay cores, showing an irregular down-core decline to steady background (supported) values below 40 cm in Central Bay and 20 cm in South Bay. Given the uncertainty in these activity profiles, an approximate chronology was determined by assuming a constant sediment flux fitted to the data by least-squares regression (the cf:cs model) (S4 Fig). Although dating uncertainty is high (± 10 years at 1950 in Central Bay; ± 22 years at 1964 in South Bay), the resulting correspondence with the known history of triclosan use and discharge (see below) provides confirmation that the dating is reasonably correct. The analysis of ^137^Cs as a supplemental dating marker was uninformative, because no radiocesium was detected below the uppermost core interval in Central Bay (0–2 cm). We attribute the absence of measurable ^137^Cs in more recent sediments to diffusional losses and/or dilution from high rates of sediment mixing.

**Fig 3:**
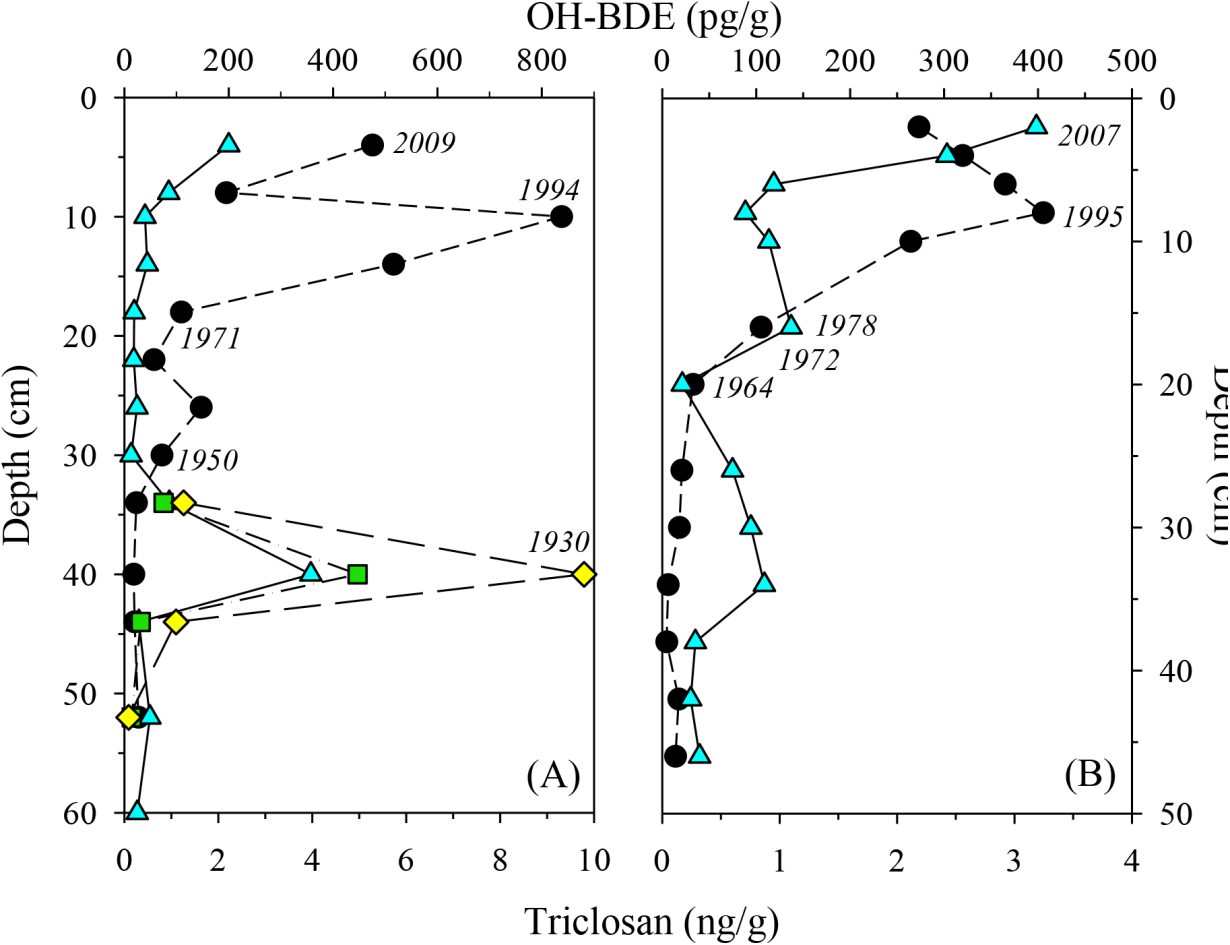
Concentration profiles of OH-BDEs and triclosan in sediment cores. (A) Central Bay, CA and (B) South Bay, CA. Profiles are given for 6-OH-BDE 47 (blue triangle), 6-OH-BDE 90 (yellow diamond), 6-OH-BDE 99 (green square), and triclosan (black circle). Italicized dates are approximate years determined by ^210^Pb.

Triclosan levels rose in Central Bay around 1960, and a maximum concentration (9.31 ng/g) was reached in ca. 1994 (Fig 3). 6’-OH-BDE 100 (1.2 ng/g) was also measured in ca. 1994 Central Bay sediment, and this was the only sample in which an anthropogenic-only-sourced OH-BDE was detected. The detection of 6’-OH-BDE 100 was determined by the presence of its un-identified, less retained transformation product during LC-MS/MS analysis. 6-OH-BDE 47 was detected throughout the sediment record with maximum concentrations in 1930 (356 pg/g) and the surface sediment (200 pg/g). Around the 1930s, 6-OH-BDE 90 and 6-OH-BDE 99 were detected at high concentrations (447 and 881 pg/g, respectively), in addition to an unknown compound, most likely another OH-PentaBDE (S1 Fig). Two additional unknown compounds, potentially OH-PentaBDEs, were detected in the two most recent sediments of Central Bay. Sediments before 1964 had low levels of triclosan (mean 0.11 ng/g, median 0.13 ng/g) in the South Bay core, and rose over time with more recent sediments having 2–3 ng/g. The occurrence of 6-OH-BDE 47 fluctuated throughout the South Bay core (concentrations ranged from 21 to 398 pg/g) with rising levels beginning in 1995 that continued to present day.

## Discussion

### Spatial Trend in Impacted vs Pristine Marine Systems

Triclosan and ∑PBDE(47 +100) were used as anthropogenic markers in this study. Wastewater effluent is the main source of triclosan, but PBDEs have numerous pathways including: manufacturing facilities [1], sewage/wastewater effluent [1,2], and atmospheric deposition [3]. The positive correlation between ∑PBDE(47 +100) and triclosan levels in San Francisco Bay suggests that these anthropogenic chemicals originate from urban sources, like wastewater effluent, and past studies have measured PBDEs in local wastewater [76].

Lower levels of the target chemicals at the BG20 and BG30 sites are most likely due to the generally lower population density, smaller and fewer nearby WWTP outfalls (Fig 1), and larger inputs of freshwater from major rivers. Natural production of OH-BDEs likely does not occur in surface waters near the outlets of the Sacramento (BG20 site) and San Joaquin River (BG30 site) because of the low salinity and bromide levels. Suisun and San Pablo Bay are also well flushed from large freshwater inflows in addition to tidal mixing [77], and are less urbanized than the central and southern bays, so lower levels of most chemical pollutants are generally expected and found [4,78]. Thus, low levels of triclosan, ∑PBDE(47 +100), biosynthetic or anthropogenic OH-BDEs, and PXDDs were detected in surface waters or sediments in the northern bays due to the low bromide levels, less urbanization, and well-flushed bays. The South and Lower South bays are not as well-flushed relative to their development density, have high salinity and bromide concentrations, and thus are more susceptible to both pollution from anthropogenic discharge and conditions favoring the accumulation of natural OH-BDEs.

Metabolites of PBDEs might be expected to have a similar distribution to PBDEs but generally at lower concentrations [79–82]. The generally uniform distribution of ∑PBDE(47 +100) (around 4- to 5-fold difference between minimum and maximum concentrations) in the southern bays was not observed for 6-OH-BDE 47 and 1,3,7-TriBDD, whose concentrations varied among sampling sites by up to 15-fold. Such uncorrelated spatial trends for these groups of chemicals were also noted along the Swedish coastline [45]. No signification correlation was seen between 6-OH-BDE 47 and ∑PBDE(47 +100), suggesting that these chemicals have different origins, most likely natural production and wastewater effluent/atmospheric deposition, respectively [45]. The lack of correlation could also be due to a variation in PBDE degradation pathways across the sampling sites in San Francisco Bay or the co-elution of an unknown OH-BDE with 6-OH-BDE 47 during LC-MS/MS analysis. Lower OH-BDE concentrations compared to PBDEs is indicative of PBDE transformation. Several studies investigating the oxidation mechanism of PBDE measured lower concentrations of OH-BDEs compared to the parent compound, owing to a slow oxidation reaction rate [79–82]. This pattern was found at most sites with 6-OH-BDE 47 lower that BDE 47, aside from SB002S and LSB042S, where the 6-OH-BDE 47 was nearly the same concentration or higher than the parent BDE 47.

On the other hand, the OH-BDEs hypothesized to be primarily anthropogenic (6’-OH-BDE 100 and 6’-OH-BDE 118), i.e. not known natural products, were not detected in San Francisco Bay (with the exception of the single detection of 6’-OH-BDE 100). The presence of 6’-OH-BDE 100 also corresponded to the maximum concentration of triclosan in the Central Bay core suggesting that at this time there may have been higher loadings of anthropogenic inputs. The overall absence of these two OH-BDEs supports the hypothesis that the dominant source of OH-BDEs in these locations is natural production. One limitation of this study is the relatively lower analytical sensitivity (1.5 to 25-fold) for 6’-OH-BDE 100 and 6’-OH-BDE 118 compared to the other OH-BDEs. Another possibility is that higher brominated PBDEs may have had slower oxidation rates, and their OH-BDE products were not detected due to the relatively higher LODs. There is a possibility, therefore, that a portion of the 6-OH-BDE 47 measured in San Francisco Bay are metabolites of BDE 47, given the previous arguments. The natural production of 6-OH-BDE 47, however, is likely more important.

Anthropogenic activities may also *indirectly* influence the natural production of OH-BDEs. The elevated nutrient load and temperature from anthropogenically impacted waters may cause a flourish in marine microbial activity near large urban areas, such as San Francisco Bay. In contrast, Point Reyes National Seashore is a lightly developed coastal ecosystem with little to no urban anthropogenic influences, which is reflected in the low levels of triclosan and 2,8-DiCDD. There is some agricultural activity (mostly ranching) in surrounding watersheds, but monitoring of nutrients in creeks draining to Point Reyes show no consistent trends of higher concentrations in watersheds with agricultural uses [83]. 6-OH-BDE 47 and 1,3,7-TriBDD levels in the national park, therefore, are representative concentrations for natural production that are slightly or negligibly altered by human activities. Slight variations in 6-OH-BDE 47 levels were observed in the samples taken from Point Reyes National Seashore (only one of the three sets of samples had levels above 10 pg/g). These concentrations, however, are smaller than or near the lowest levels measured pre-1970s sediments in the South Bay (21–108 pg/g) and Central Bay (13–86 pg/g, excluding the ca. 1930 spike) cores. Therefore, the overall higher 6-OH-BDE 47 concentrations observed in the urbanized San Francisco estuary suggest that enhanced natural production (or, less likely, degradation of anthropogenic PBDEs) is occurring in the estuary.

### Freshwater vs Coastal Systems

Lake Pepin is a natural impoundment of the upper Mississippi River, located downstream of several WWTPs and the large metropolitan centers of Minneapolis and St. Paul, MN. Buth et al [62] documented the historical accumulation of triclosan and its triclosan-derived dioxin in Lake Pepin since the 1960s. Although no previous studies have investigated PBDE levels in Lake Pepin, PBDEs are ubiquitous and it is highly likely that PBDEs are present in Lake Pepin sediments given the notable presence of triclosan. Because natural production of OH-BDEs cannot occur in freshwater, any OH-BDEs present in Lake Pepin are likely derived from PBDEs. No OH-BDEs were detected and there were only low levels of 1,3,7-TriBDD in a few samples. The presence of 1,3,7-TriBDD without detected 6-OH-BDE 47 could be due to a lower detection limit for the PBDD, or the brominated dioxin was a combustion product of brominated flame retardants and atmospherically deposited from regional sources. Other researchers have found chlorinated dioxins in freshwater sediments, but no brominated dioxins, which indicate different sources [44], or at least different relative magnitudes of their sources. Chlorine is naturally more abundant, with measured PCDD formation even from combustion of wood and other natural fuels, whereas literature on combustion formed PBDDs primarily documents production from co-combustion of anthropogenic wastes, which generally include PBDEs. This is not to say, however, that there is no PBDD production from combustion of natural fuels, given potentially trace levels of bromines in many materials. Some PBDD formation from combustion is possible and perhaps even likely, but may be too low to measure using current analytical methods.

### Temporal Trends

Although radiometric dating of the two coastal cores from San Francisco Bay yielded results with substantial uncertainties, the resulting chronologies agree well with the expected temporal trends of triclosan, providing important confirmation of the historical trend of OH-BDE production. As seen in ^210^Pb, the profile of triclosan may have been affected by fluctuating sedimentation rates which caused the drop in Central Bay after 1994. The noticeable rising levels after the 1960s, however, confirm the overall usage trends for this anthropogenic chemical. The presence of triclosan near the LOD in pre-1960s sediments is likely due to contamination during the collection, extraction, and/or sample clean-up process. It is extremely difficult to maintain a triclosan-free laboratory environment due to the ubiquitous presence of triclosan. Levels of triclosan pre-1960 are much lower than post-1960s, and thus samples were not significantly contaminated.

Based on the presence of OH-BDEs throughout the cores, it is likely that these compounds were naturally produced in Central and South Bay during the last century. 6-OH-BDE 47 is present throughout both cores, even though PBDEs were not used in consumer products until the 1970s. The accumulation of OH-BDEs in the environment, however, is likely influenced by other anthropogenic drivers. Around the 1930s, concentrations of the biosynthesized 6-OH-BDE 47, 6-OH-BDE 90, and 6-OH-BDE 99 in Central Bay spiked upward, and an unidentified, probably biosynthetic, OH-PentaBDE was detected. Increasing population and the discharge of raw sewage into San Francisco Bay may have contributed to enhanced natural production [84]. It was roughly estimated that in 1910 approximately half of the total population in California was disposing of untreated sewage by discharging into estuaries, tidal bays, etc [85]. It was not until the early 1950s that San Jose, San Francisco, Oakland and other communities surrounding the bay built primary wastewater treatment plants to combat and resolve the pollution of the bay [84]. The drought that occurred from 1928–1934 may have also influenced OH-BDEs production in the system. With the exception of Central Bay in the 1930s, which was more heavily developed at the time, the levels of 6-OH-BDE 47 pre-1970s in the rest of the bay were similar to those measured in Point Reyes National Seashore, which is consistent with the expectation that enhanced biosynthesis would occur in the more developed areas.

The increasing levels of 6-OH-BDE 47 after the 1970s are likely a result of increased natural production. That is not to say that the transformation from PBDEs via abiotic (i.e. photolysis and oxidation by OH radicals) and biotic processes (i.e. metabolic oxidation) cannot account for a percentage of the more recent rise and fall in levels in San Francisco Bay, but our work suggests that biogenic production enhanced by wastewater discharge, another anthropogenic activity, or potentially even climate change are more likely contributors given: (1) the lack of synthetic PBDEs during an earlier ca. 1930s spike in OH-BDEs; (2) no OH-BDEs were detected in the anthropogenically impacted freshwater lakes; and (3) lower OH-BDE levels were measured in the relatively pristine marine system than in surface sediments from the anthropogenically impacted bay. Rising levels of PBDDs in mussels over the past decade was attributed to eutrophication and climate change enhancing biosynthetic production [44]. Thus, the OH-BDE producers, e.g. marine bacteria, may flourish in waters with high nutrient levels and temperatures, and these conditions are believed to enhance the natural production of OH-BDEs and PBDDs.

## Supporting Information

S1 Appendix Extraction and analytical methods. (PDF)

S2 Appendix Cleaning protocols and calculation of analyte concentrations. (PDF)

S1 Fig Representative chromatograms using LC-MS/MS method displaying SRM transitions and retention times for: (A) a standard; and (B) Central Bay 38–40 cm sediment. (PDF)

S2 Fig Loss-on-ignition results for: (A) Central Bay (CB001S) core, and (B) South Bay (SB002S) core. (PDF)

S3 Fig Graphs displaying a: (A) significant correlation between ∑PBDE(47+100) and triclosan (TCS); (B) insignificant correlation between 6-OH-BDE 47 and ∑PBDE(47+100); (C) insignificant correlation between triclosan and 6-OH-BDE 47; (D) significant correlation between 6-OH-BDE 47 and 1,3,7-TriBDD; and (E) significant correlation between triclosan and its dioxin (2,8-DiCDD) in San Francisco Bay surface sediments. (PDF)

S4 Fig An approximate chronology was determined for the San Francisco Bay cores by assuming a constant sediment flux (DMAR; dry mass accumulation rate) fitted to the data by least-squares regression (the cf:cs model). (PDF)

S1 Table Selected reaction monitoring transitions (SRM) for chemical quantification (Q) and confirmation (C). (PDF)

S2 Table Absolute and relative recovery for ^13^C_12_-PXDDs and PXDDs, respectively, in sediments. (PDF)

S3 Table Loss-on-ignition results for San Francisco Bay surface sediments and Point Reyes National Seashore cores. (PDF)

S4 Table Latitude and longitude of salinity measurements for surface water sampling sites near the sediment sampling sites. (PDF)

## References

[1] Hale RC, Alaee M, Manchester-Neesvig JB, Stapleton HM, Ikonomou MG. Polybrominated diphenyl ether flame retardants in the North American environment. Environ Int. 2003 Sep;29(6):771–779. 12850095 10.1016/S0160-4120(03)00113-2

[2] North KD. Tracking polybrominated diphenyl ether releases in a wastewater treatment plant effluent, Palo Alto, California. Environ Sci Technol. 2004 9;38(17):4484–4488. 15461153 10.1021/es049627y

[3] Hale RC, La Guardia MJ, Harvey E, Mainor TM. Potential role of fire retardant-treated polyurethane foam as a source of brominated diphenyl ethers to the US environment. Chemosphere. 2002 2;46(5):729–735. 11999796 10.1016/s0045-6535(01)00237-5

[4] Oros D, Hoover D, Rodigari F, Crane D, Sericano J. Levels and distribution of polybrominated diphenyl ethers in water, surface sediments, and bivalves from the San Francisco Estuary. Environ Sci Technol. 2005 1;39(1):33–41. 15667072 10.1021/es048905q

[5] Sutton R, Sedlak MD, Yee D, Davis JA, Crane D, Grace R, et al Declines in Polybrominated Diphenyl Ether Contamination of San Francisco Bay following Production Phase-Outs and Bans. Environ Sci Technol. 2015 1;49(2):777–784. 10.1021/es503727b 25544014

[6] Marsh G, Athanasiadou M, Bergman A, Asplund L. Identification of hydroxylated and methoxylated polybrominated diphenyl ethers in Baltic Sea salmon (Salmo salar) blood. Environ Sci Technol. 2004 1;38(1):10–18. 14740711 10.1021/es034671j

[7] Erratico CA, Moffatt SC, Bandiera SM. Comparative Oxidative Metabolism of BDE-47 and BDE-99 by Rat Hepatic Microsomes. Toxicol. Sci. 2011 9;123(1):37–47. 10.1093/toxsci/kfr155 21673328

[8] Ueno D, Darling C, Alaee M, Pacepavicius G, Teixeira C, Campbell L, et al Hydroxylated Polybrominated diphenyl ethers (OH-PBDEs) in the abiotic environment: Surface water and precipitation from Ontario, Canada. Environ Sci Technol. 2008 3;42(5):1657–1664. 18441817 10.1021/es7021279

[9] Malmvarn A, Marsh G, Kautsky L, Athanasiadou M, Bergman A, Asplund L. Hydroxylated and methoxylated brominated diphenyl ethers in the red algae Ceramium tenuicorne and blue mussels from the Baltic Sea. Environ Sci Technol. 2005 5;39(9):2990–2997. 15926543 10.1021/es0482886

[10] Hua W, Bennett E, Letcher R. Triclosan in waste and surface waters from the upper Detroit River by liquid chromatography-electrospray-tandem quadrupole mass spectrometry. Environ Int. 2005 7;31(5):621–630. 15910958 10.1016/j.envint.2004.10.019

[11] Liu H, Zhao H, Quan X, Zhang Y, Chen S, Zhao H. Formation of 2 '-hydroxy-2,3 ',4,5 '-tetrabromodipheyl ether (2 '-HO-BDE68) from 2,4-dibromophenol in aqueous solution under simulated sunlight irradiation. Chemosphere. 2011 7;84(4):512–518. 10.1016/j.chemosphere.2011.03.011 21459404

[12] Zhao Q, Zhao H, Quan X, He X, Chen S. Photochemical Formation of Hydroxylated Polybrominated Diphenyl Ethers (OH-PBDEs) from Polybrominated Diphenyl Ethers (PBDEs) in Aqueous Solution under Simulated Solar Light Irradiation. Environ Sci Technol. 2015 7;49(15):9092–9099. 10.1021/acs.est.5b01240 26134578

[13] Malmvarn A, Zebuhr Y, Kautsky L, Bergman A, Asplund L. Hydroxylated and methoxylated polybrominated diphenyl ethers and polybrominated dibenzo-p-dioxins in red alga and cyanobacteria living in the Baltic Sea. Chemosphere. 2008 6;72(6):910–916. 10.1016/j.chemosphere.2008.03.036 18457860

[14] Agarwal V, El Gamal AA, Yamanaka K, Poth D, Kersten RD, Schorn M, et al Biosynthesis of polybrominated aromatic organic compounds by marine bacteria. Nat. Chem. Biol. 2014 8;10(8):640–U182. 10.1038/nchembio.1564 24974229 PMC4104138

[15] Anjaneyulu V, Rao K, Radhika P, Muralikrishna M, Connolly J. A new tetrabromodiphenyl ether from the sponge Dysidea herbacea of the Indian Ocean. Indian J. Chem., Sect B. 1996 1;35(1):89–90.

[16] Cameron G, Stapleton B, Simonsen S, Brecknell D, Garson M. New sesquiterpene and brominated metabolites from the tropical marine sponge Dysidea sp. Tetrahedron. 2000 7;56(29):5247–5252.

[17] Lofstrand K, Liu X, Lindqvist D, Jensen S, Asplund L. Seasonal variations of hydroxylated and methoxylated brominated diphenyl ethers in blue mussels from the Baltic Sea. Chemosphere. 2011 7;84(4):527–532. 10.1016/j.chemosphere.2011.01.001 21288551

[18] Hensley RN, Kerrigan JF, Pang H, Erickson PR, Grandbois M, McNeill K, et al Triclosan, Chlorinated Triclosan Derivatives, and Hydroxylated Polybrominated Diphenyl Ethers (OH-BDEs) in Wastewater Effluents. Environ Sci: Water Res. 2015 2;1:316–325.

[19] Lin K, Yan C, Gan J. Production of Hydroxylated Polybrominated Diphenyl Ethers (OH-PBDEs) from Bromophenols by Manganese Dioxide. Environ Sci Technol. 2014 1;48(1):263–271. 10.1021/es403583b 24266690

[20] Agarwal V, Li J, Rahman I, Borgen M, Aluwihare LI, Biggs JS, et al Complexity of Naturally Produced Polybrominated Diphenyl Ethers Revealed via Mass Spectrometry. Environ Sci Technol. 2015 2;49(3):1339–46. 10.1021/es505440j 25559102 PMC4358748

[21] Unson M, Holland N, Faulkner D. A Brominated Secondary Metabolite Synthesized by the Cyanobacterial Symbiont of a Marine Sponge and Accumulation of the Crystalline Metabolite in the Sponge Tissue. Mar Biol. 1994 4;119(1):1–11.

[22] Handayani D, Edrada R, Proksch P, Wray V, Witte L, Van Soest R, et al Four new bioactive polybrominated diphenyl ethers of the sponge Dysidea herbacea from west Sumatra, Indonesia. J Nat Prod. 1997 12;60(12):1313–1316. 9463111 10.1021/np970271w

[23] Asplund L, Athanasiadou M, Sjodin A, Bergman A, Borjeson H. Organohalogen substances in muscle, egg and blood from healthy Baltic salmon (Salmo salar) and Baltic salmon that produced offspring with the M74 syndrome. Ambio. 1999 2;28(1):67–76.

[24] Wan Y, Wiseman S, Chang H, Zhang X, Jones PD, Hecker M, et al Origin of Hydroxylated Brominated Diphenyl Ethers: Natural Compounds or Man-Made Flame Retardants? Environ Sci Technol. 2009 10;43(19):7536–7542. 19848173 10.1021/es901357u

[25] McKinney MA, Cesh LS, Elliott JE, Williams TD, Garcelon DK, Letcher RJ. Brominated flame retardants and halogenated phenolic compounds in North American west coast bald eaglet (Haliaeetus leucocephalus) plasma. Environ Sci Technol. 2006 10;40(20):6275–6281. 17120553 10.1021/es061061l

[26] Hovander L, Malmberg T, Athanasiadou M, Athanassiadis L, Rahm S, Bergman A, et al Identification of hydroxylated PCB metabolites and other phenolic halogenated pollutants in human blood plasma. Arch Environ Contam Toxicol. 2002 1;42(1):105–117. 11706375 10.1007/s002440010298

[27] Zhang K, Wan Y, Jones PD, Wiseman S, Giesy JP, Hu J. Occurrences and Fates of Hydroxylated Polybrominated Diphenyl Ethers in Marine Sediments in Relation to Trophodynamics. Environ Sci Technol. 2012 2;46(4):2148–2155. 10.1021/es203195s 22296595

[28] Fan Y, Lan J, Li H, Li G, Cao Y, Zhao Z, et al Spatial distributions of methoxylated and hydroxylated polybrominated diphenyl ethers in the East China Sea A seaward increasing trend. Chemosphere. 2014 11;114:247–254. 10.1016/j.chemosphere.2014.04.103 25113209

[29] Kim U, Nguyen Thi Hoang Yen, Oh J. Hydroxylated, Methoxylated, and Parent Polybrominated Diphenyl Ethers (PBDEs) in the Inland Environment, Korea, and Potential OH- and MeO-BDE Source. Environ Sci Technol. 2014 7;48(13):7245–7253. 10.1021/es5006972 24911666

[30] Legler J. New insights into the endocrine disrupting effects of brominated flame retardants. Chemosphere. 2008 9;73(2):216–222. 10.1016/j.chemosphere.2008.04.081 18667224

[31] Dingemans MML, de Groot A, van Kleef RGDM, Bergman A, van den Berg M, Vijverberg HPM, et al Hydroxylation increases the neurotoxic potential of BDE-47 to affect exocytosis and calcium homeostasis in PC12 cells. Environ Health Perspect. 2008 5;116(5):637–643. 10.1289/ehp.11059 18470311 PMC2367675

[32] Van Boxtel AL, Kamstra JH, Cenijn PH, Pieterse B, Wagner MJ, Antink M, et al Microarray analysis reveals a mechanism of phenolic polybrominated diphenylether toxicity in zebrafish. Environ Sci Technol. 2008 3;42(5):1773–1779. 18441834 10.1021/es0720863

[33] Legradi J, Dahlberg A, Cenijn P, Marsh G, Asplund L, Bergman A, et al Disruption of Oxidative Phosphorylation (OXPHOS) by Hydroxylated Polybrominated Diphenyl Ethers (OH-PBDEs) Present in the Marine Environment. Environ Sci Technol. 2014 12;48(24):14703–14711. 10.1021/es5039744 25422162

[34] Lai Y, Lu M, Lin S, Cai Z. Glucuronidation of hydroxylated polybrominated diphenyl ethers and their modulation of estrogen UDP-glucuronosyltransferases. Chemosphere. 2012 2;86(7):727–734. 10.1016/j.chemosphere.2011.10.047 22119418

[35] Zota AR, Park J, Wang Y, Petreas M, Zoeller RT, Woodruff TJ. Polybrominated Diphenyl Ethers, Hydroxylated Polybrominated Diphenyl Ethers, and Measures of Thyroid Function in Second Trimester Pregnant Women in California. Environ Sci Technol. 2011 9;45(18):7896–7905. 10.1021/es200422b 21830753 PMC3191110

[36] Ucan-Marin F, Arukwe A, Mortensen A, Gabrielsen GW, Fox GA, Letcher RJ. Recombinant Transthyretin Purification and Competitive Binding with Organohalogen Compounds in Two Gull Species (Larus argentatus and Larus hyperboreus). Toxicol. Sci. 2009 2;107(2):440–450. 10.1093/toxsci/kfn240 19033396

[37] Erickson PR, Grandbois M, Arnold WA, McNeill K. Photochemical Formation of Brominated Dioxins and Other Products of Concern from Hydroxylated Polybrominated Diphenyl Ethers (OH-PBDEs). Environ Sci Technol. 2012 8;46(15):8174–8180. 10.1021/es3016183 22765251

[38] Arnoldsson K, Andersson PL, Haglund P. Photochemical Formation of Polybrominated Dibenzo-p-dioxins from Environmentally Abundant Hydroxylated Polybrominated Diphenyl Ethers. Environ Sci Technol. 2012 7;46(14):7567–7574. 10.1021/es301256x 22721005

[39] Buser HR. Polybrominated Dibenzofurans and Dibenzo-Para-Dioxins—Thermal-Reaction Products of Polybrominated Diphenyl Ether Flame Retardants. Environ Sci Technol. 1986 4;20(4):404–408. 10.1021/es00146a015 22300214

[40] Sovocool GW, Mitchum RK, Tondeur Y, Munslow WD, Vonnahme TL, Donnelly JR. Bromo-Polynuclear and Bromochloro-Polynuclear Aromatic-Hydrocarbons, Dioxins and Dibenzofurans in Municipal Incinerator Fly-Ash. Biomed. Environ. Mass Spectrom. 1988 6;15(12):669–676. 3416090 10.1002/bms.1200151205

[41] Tu L, Wu Y, Wang L, Chang-Chien G. Distribution of Polybrominated Dibenzo-p-dioxins and Dibenzofurans and Polybrominated Diphenyl Ethers in a Coal-fired Power Plant and Two Municipal Solid Waste Incinerators. Aerosol Air Qual. Res. 2011 10;11(5):596–615.

[42] Soderstrom G, Marklund S. PBCDD and PBCDF from incineration of waste-containing brominated flame retardants. Environ Sci Technol. 2002 5;36(9):1959–1964. 12026978 10.1021/es010135k

[43] Wang L, Chang-Chien G. Characterizing the emissions of polybrominated dibenzo-p-dioxins and dibenzofurans from municipal and industrial waste incinerators. Environ Sci Technol. 2007 2;41(4):1159–1165. 17593714 10.1021/es061801q

[44] Haglund P, Malmvarn A, Bergek S, Bignert A, Kautsky L, Nakano T, et al Brominated dibenzo-p-dioxins: A new class of marine toxins? Environ Sci Technol. 2007 5;41(9):3069–3074. 17539506 10.1021/es0624725

[45] Lofstrand K, Malmvarn A, Haglund P, Bignert A, Bergman A, Asplund L. Brominated phenols, anisoles, and dioxins present in blue mussels from the Swedish coastline. Environ. Sci. Pollut. R. 2010 9;17(8):1460–1468. 10.1007/s11356-010-0331-1 20396970

[46] Unger M, Asplund L, Haglund P, Malmvarn A, Arnoldsson K, Gustafsson O. Polybrominated and Mixed Brominated/Chlorinated Dibenzo-p-Dioxins in Sponge (Ephydatia fluviatilis) from the Baltic Sea. Environ Sci Technol. 2009 11;43(21):8245–8250. 10.1021/es901705r 19924951

[47] Agarwal V, Moore BS. Enzymatic Synthesis of Polybrominated Dioxins from the Marine Environment. ACS Chem Biol. 2014 9;9(9):1980–1984. 10.1021/cb5004338 25061970 PMC4168793

[48] Mason G, Zacharewski T, Denomme MA, Safe L, Safe S. Polybrominated Dibenzo-Para-Dioxins and Related-Compounds—Quantitative Invivo and Invitro Structure-Activity-Relationships. Toxicology. 1987 6;44(3):245–255. 3033849 10.1016/0300-483x(87)90027-8

[49] Birnbaum L, Staskal D, Diliberto J. Health effects of polybrominated dibenzo-p-dioxins (PBDDs) and dibenzofurans (PBDFs). Environ Int. 2003 9;29(6):855–860. 12850101 10.1016/S0160-4120(03)00106-5

[50] van den Berg M, Denison MS, Birnbaum LS, DeVito MJ, Fiedler H, Falandysz J, et al Polybrominated Dibenzo-p-Dioxins, Dibenzofurans, and Biphenyls: Inclusion in the Toxicity Equivalency Factor Concept for Dioxin-Like Compounds. Toxicol Sci. 2013 6;133(2):197–208. 10.1093/toxsci/kft070 23492812 PMC3663561

[51] Mason G, Farrell K, Keys B, Piskorskapliszczynska J, Safe L, Safe S. Polychlorinated Dibenzo-Para-Dioxins—Quantitative Invitro and Invivo Structure-Activity-Relationships. Toxicology. 1986 10;41(1):21–31. 3750336 10.1016/0300-483x(86)90101-0

[52] Latch D, Packer J, Arnold W, McNeill K. Photochemical conversion of triclosan to 2,8-dichlorodibenzo-p-dioxin in aqueous solution. J Photochem Photobiol A. 2003 5;158(1):63–66.

[53] Latch DE, Packer JL, Stender BL, VanOverbeke J, Arnold WA, McNeill K. Aqueous photochemistry of triclosan: Formation of 2,4-dichlorophenol, 2,8-dichlorodibenzo-p-dioxin, and oligomerization products. Environ Toxicol Chem. 2005 3;24(3):517–525. 15779749 10.1897/04-243r.1

[54] Ciba Specialty Chemical. Irgasanrˋ DP 300, Irgacarerˋ MP. Toxicological and Ecological Data. Official Registrations. 1998.

[55] McAvoy D, Schatowitz B, Jacob M, Hauk A, Eckhoff W. Measurement of triclosan in wastewater treatment systems. Environ Toxicol Chem. 2002 7;21(7):1323–1329. 12109730

[56] Aguera A, Fernandez-Alba A, Piedra L, Mezcua M, Gomez M. Evaluation of triclosan and biphenylol in marine sediments and urban wastewaters by pressurized liquid extraction and solid phase extraction followed by gas chromatography mass spectrometry and liquid chromatography mass spectrometry. Anal Chim Acta. 2003 3;480(2):193–205.

[57] Lishman L, Smyth SA, Sarafin K, Kleywegt S, Toito J, Peart T, et al Occurrence and reductions of pharmaceuticals and personal care products and estrogens by municipal wastewater treatment plants in Ontario, Canada. Sci Total Environ. 2006 8;367(2–3):544–558. 16697441 10.1016/j.scitotenv.2006.03.021

[58] Stasinakis AS, Gatidou G, Mamais D, Thomaidis NS, Lekkas TD. Occurrence and fate of endocrine disrupters in Greek sewage treatment plants. Water Res. 2008 3;42(6–7):1796–1804. 18048079 10.1016/j.watres.2007.11.003

[59] Buth JM, Ross MR, McNeill K, Arnold WA. Removal and formation of chlorinated triclosan derivatives in wastewater treatment plants using chlorine and UV disinfection. Chemosphere. 2011 8;84(9):1238–1243. 10.1016/j.chemosphere.2011.05.017 21652055

[60] Ying G, Kookana RS. Triclosan in wastewaters and biosolids from Australian wastewater treatment plants. Environ Int. 2007 2;33(2):199–205. 17055058 10.1016/j.envint.2006.09.008

[61] Anger CT, Sueper C, Blumentrit DJ, McNeill K, Engstrom DR, Arnold WA. Quantification of Triclosan, Chlorinated Triclosan Derivatives, and their Dioxin Photoproducts in Lacustrine Sediment Cores. Environ Sci Technol. 2013 2;47(4):1833–1843. 10.1021/es3045289 23320506

[62] Buth JM, Steen PO, Sueper C, Blumentritt D, Vikesland PJ, Arnold WA, et al Dioxin Photoproducts of Triclosan and Its Chlorinated Derivatives in Sediment Cores. Environ Sci Technol. 2010 6;44(12):4545–4551. 10.1021/es1001105 20476764

[63] Venkatesan AK, Pycke BFG, Barber LB, Lee KE, Halden RU. Occurrence of triclosan, triclocarban, and its lesser chlorinated congeners in Minnesota freshwater sediments collected near wastewater treatment plants. J Hazard Mater. 2012 8;229:29–35. 10.1016/j.jhazmat.2012.05.049 22742731 PMC3401314

[64] Kolpin D, Furlong E, Meyer M, Thurman E, Zaugg S, Barber L, et al Pharmaceuticals, hormones, and other organic wastewater contaminants in US streams, 1999–2000: A national reconnaissance. Environ Sci Technol. 2002 3;36(6):1202–1211. 11944670 10.1021/es011055j

[65] Jacobs M, Nolan G, Hood S. Lignans, bacteriocides and organochlorine compounds activate the human pregnane X receptor (PXR). Toxicol Appl Pharmacol. 2005 12;209(2):123–133. 15885729 10.1016/j.taap.2005.03.015

[66] DeLorenzo ME, Keller JM, Arthur CD, Finnegan MC, Harper HE, Winder VL, et al Toxicity of the antimicrobial compound triclosan and formation of the metabolite methyl-triclosan in estuarine systems. Environ Toxicol. 2008 4;23(2):224–232. 10.1002/tox.20327 18214910

[67] Perron MM, Ho KT, Cantwell MG, Burgess RM, Pelletier MC. Effects of triclosan on marine benthic and epibenthic organisms. Environ Toxicol Chem. 2012 8;31(8):1861–1866. 10.1002/etc.1884 22605471

[68] Dann AB, Hontela A. Triclosan: environmental exposure, toxicity and mechanisms of action. J Appl Toxicol. 2011 5;31(4):285–311. 10.1002/jat.1660 21462230

[69] Steen PO, Grandbois M, McNeill K, Arnold WA. Photochemical Formation of Halogenated Dioxins from Hydroxylated Polybrominated Diphenyl Ethers (OH-PBDEs) and Chlorinated Derivatives (OH-PBCDEs). Environ Sci Technol. 2009 6;43(12):4405–4411. 19603654 10.1021/es9003679

[70] Appleby P. Chronostratigraphic techniques in recent sediments In: Last WM, Smol JP, editors. Tracking Environmental Change Using Lake Sediments: Basin Analysis, Coring, And Chronological Techniques, Eds Kluwer Academic Publishers: Dordrecht; 2002 p. 171–203.

[71] Eakins JD, Morrison RT. New Procedure for Determination of Pb-210 in Lake and Marine-Sediments. Int J Appl Radiat Isot. 1978;29(9–10):531–536.

[72] Blumentritt DJ, Engstrom DR, Balogh SJ. A novel repeat-coring approach to reconstruct recent sediment, phosphorus, and mercury loading from the upper Mississippi River to Lake Pepin, USA. J Paleolimnol. 2013 10;50(3):293–304.

[73] U.S. EPA. Method 1613 Tetra- through Octa-Chlorinated Dioxins and Furans by Isotope Dilution HRGC/HRMS. 1994.

[74] Luisa Feo M, Baron E, Aga DS, Eljarrat E, Barcelo D. Development of a liquid chromatography-electrospray chemical ionization tandem mass spectrometry analytical method for analysis of eleven hydroxylated polybrominated diphenyl ethers. J Chromatogr A. 2013 8;1301:80–87. 10.1016/j.chroma.2013.05.062 23791149

[75] San Francisco Estuary Institute. Contaminant Data Display & Download. Available at: http://www.sfei.org/rmp/wqt. Accessed August, 2014.

[76] North K. Tracking polybrominated diphenyl ether releases in a wastewater treatment plant effluent, Palo Alto, California. Environ Sci Technol. 2004 9;38(17):4484–4488. 15461153 10.1021/es049627y

[77] Rodenburg LA, Meng Q, Yee D, Greenfield BK. Evidence for photochemical and microbial debromination of polybrominated diphenyl ether flame retardants in San Francisco Bay sediment. Chemosphere. 2014 Jul;106:36–43. 10.1016/j.chemosphere.2013.12.083 24485321

[78] Klosterhaus SL, Stapleton HM, La Guardia MJ, Greig DJ. Brominated and chlorinated flame retardants in San Francisco Bay sediments and wildlife. Environ Int. 2012 10;47:56–65. 10.1016/j.envint.2012.06.005 22766500

[79] Valters K, Li H, Alaee M, D'Sa I, Marsh G, Bergman A, et al Polybrominated diphenyl ethers and hydroxylated and methoxylated brominated and chlorinated analogues in the plasma of fish from the Detroit River. Environ Sci Technol. 2005 8;39(15):5612–5619. 16124294 10.1021/es0506410

[80] Stapleton HM, Eagle S, Anthopolos R, Wolkin A, Miranda ML. Associations between Polybrominated Diphenyl Ether (PBDE) Flame Retardants, Phenolic Metabolites, and Thyroid Hormones during Pregnancy. Environ Health Perspect. 2011 10;119(10):1454–1459. 10.1289/ehp.1003235 21715241 PMC3230439

[81] Athanasiadou M, Cuadra SN, Marsh G, Bergman A, Jakobsson K. Polybrominated diphenyl ethers (PBDEs) and bioaccumulative hydroxylated PBDE metabolites in young humans from Managua, Nicaragua. Environ Health Perspect. 2008 3;116(3):400–408. 18335110 10.1289/ehp.10713 PMC2265063

[82] Routti H, Letcher RJ, Arukwe A, van Bavel B, Yoccoz NG, Chu S, et al Biotransformation of PCBs in Relation to Phase I and II Xenobiotic-Metabolizing Enzyme Activities in Ringed Seals (Phoca hispida) from Svalbard and the Baltic Sea. Environ Sci Technol. 2008 12;42(23):8952–8958. 19192824 10.1021/es801682f

[83] Anima RJ. Pollution Studies of Drakes Estero, and Abbotts Lagoon Point Reyes National Seashore, California, USA. 1991;Open File Report 91–145.

[84] Sedlak D. Water 4.0: the past, present, and future of the world's most vital resource New Haven & London: Yale University Press; 2014.

[85] Hyde CH. Stream Pollution and Present Status of Controlling Legislation in California. Am J Public Health. 1914;4(10). 10.2105/ajph.4.10.819-a PMC1286441 18009103

