# Supplementary material for: Quantification of Hydroxylated Polybrominated Diphenyl Ethers (OH-BDEs), Triclosan, and Related Compounds in Freshwater and Coastal Systems
Source: PLoS One. 2015 Oct 14;10(10):e0138805. doi: 10.1371/journal.pone.0138805 (PMC4605494; doi:10.1371/journal.pone.0138805)
Supplement: S1 Appendix — (PDF) [file pone.0138805.s001.pdf]

S1 Appendix

Hydroxylated Polybrominated Diphenyl Ethers (OH-BDEs) Surface Water and Sediment Extraction Methods.

Surface water. Four replicate samples (1.5 L) were extracted for each water sample. Triplicate spike and recovery samples and method blanks were done in surface waters (0.5 L) and ultrapure water (0.5 L), respectively. All replicates were spiked with isotope labeled compounds, $^{13}C_{12}$-TCS (0.5 nM), $^{13}C_{12}$-6-OH-BDE 47 (0.6 nM), $^{13}C_{12}$-6'-OH-BDE 100 (0.5 nM), which served as surrogates and internal standards. Recovery samples were also spiked with triclosan (1 nM), 6-OH-BDE 47 (0.3 nM), 6-OH-BDE 90 (0.4 nM), 6-OH-BDE 99 (0.4 nM), 6'-OH-BDE 100 (0.3 nM), and 6'-OH-BDE 118 (0.3 nM). Samples were covered with foil and equilibrated overnight. Chemicals were extracted with an offline solid phase extraction (SPE) manifold and Waters Oasis HLB (6 cc/200 mg) cartridges. Cartridges were preconditioned with 6 mL of MTBE, MeOH, and pH 3 H_2O. Samples were loaded onto the column using vacuum pressure at a flow rate of ~15 mL/min. The flow rate was controlled by placing a sample on a balance and monitoring its decreasing mass. After the water sample was loaded, the cartridge was triple rinsed with 50:50 H_2O:MeOH (6 mL), dried by using a vacuum (15 min) to pull air through the cartridge, and eluted with MeOH (10 mL) and 90:10 MTBE:MeOH (5 mL). The extract was blown down under nitrogen to ~ 500 µL.

Each silica column was prepared in a plastic syringes (6 cc) with a small plug of glass wool at the outlet followed by a thin layer of sand, silica gel (~2 g), and another thin layer of sand at the top. Silica gel was added via an ethyl acetate slurry. The silica column was primed with ethyl acetate (10 mL) to remove any possible contaminants. Next, the extract was quantitatively added to a column and eluted with ethyl acetate (13 mL). The collected eluent was blown down to dryness under nitrogen, re-suspended in 50:50 H_2O:acetonitrile, and transferred

(180 µL) to a vial for LC-MS/MS analysis. The BG30 and LSB055W surface waters had a concentration factor of 20,000 and 7,500, respectively, whereas the recovery and method blank samples were concentrated by 2,500-fold.

Sediment. Freeze dried sediments (10 g) were spiked with $^{13}C_{12}$-TCS (60 ng), $^{13}C_{12}$-6-OH-BDE 47 (300 ng), and $^{13}C_{12}$-6'-OH-BDE 100 (300 ng). Sediments from the bottom of San Francisco Bay and Point Reyes National Seashore cores were used for recovery analysis. Triplicate recovery samples were also spiked with 6-OH-BDE 47 (100 ng), 6-OH-BDE 90 (12 ng), 6-OH-BDE 99 (12 ng), 6'-OH-BDE 100 (100 ng), 6'-OH-BDE 118 (12 ng), and triclosan (20 ng), which were dissolved in acetonitrile (100 µL). Spiked samples were covered with foil and equilibrated overnight.

The ASE cells (stainless steel, 22 mL) were prepared with two glass fiber filters on bottom followed by a thin sand layer, freeze-dried sediment, thin sand layer, and 1 glass fiber filter. Additional sand was mixed in with the sediment using a disposable glass pipette if sediment volume was not sufficient to fill the cell body. The ASE method used was: temperature: 100 °C, pressure: 1500 psi; cell heat time: 5 min; cell static time: 5 min; rinse volume: 100 %; purge time: 100 s; extraction cycles: 2; and solvent: dichloromethane. Approximately 20% of the extract was blown down to dryness with nitrogen and re-suspended (~500 µL) in the same mixture as the SPE eluent (55:45 MeOH:MTBE). The sediment extract was cleaned using the silica column method described above.

Polychlorinated dibenzo-p-dioxin (PCDD), Polychlorinated dibenzofuran (PCDF) and Polybrominated dibenzo-p-dioxin (PBDD) Extraction Method

Once spiked with labeled recovery surrogates, each sample was extracted with toluene for at least 18 hours using a Soxhlet/Dean Stark apparatus. Extracts were subsequently spiked with $^{37}Cl_4$-2,3,7,8-TCDD to measure the efficiency of sample cleanup. Soxhlet/Dean Stark

extracts were concentrated using a Snyder column, back-extracted with concentrated H_2SO_4 and NaOH, and eluted through multi-layer silica columns (2 g neutral silica, 4 g acidic silica, and 2 g basic silica) with hexane. Eluates were then added to 4 g activated aluminum oxide (Al_2O_3) columns and eluted with 60:40 DCM:hexane (v/v). After solvent exchange into hexane, Al_2O_3 column eluates were cleaned up via carbon chromatography, where samples were passed through 0.5 g of 18% activated carbon mixed with Celite. These columns were preconditioned with 5 mL of toluene, 2 mL of 75:20:5 DCM:MeOH:toluene (v/v/v), 2 mL of 50:50 DCM:cyclohexane (v/v), and 5 mL of hexane. Sample extracts were added to the column and flushed in the forward direction with 2 mL of 50:50 DCM:cyclohexane (v/v) and 2 mL of 75:20:5 DCM:MeOH:toluene (v/v/v) to remove potential interfering compounds. Finally, analytes were washed off the column in the reverse direction with 10 mL of toluene. The toluene was then concentrated, spiked with $^{13}C_{12}$-1,2,3,4-TCDD and $^{13}C_{12}$-1,2,3,7,8,9-HxCDD as recovery standards, and concentrated to a final volume of 20 μL.

HRGC-HRMS Analysis, Analyte Quantification, and QA/QC

PCDD/F and PBDD analyses were performed using high-resolution gas chromatography-high-resolution mass spectrometry (HRGC-HRMS). Aliquots of final extracts (1 μL) were injected into an HP 5890 gas chromatograph with a split/splitless injector and a 60 meter DB-5MS capillary column (0.25 mm ID x 0.25 μm film). An additional analysis substituting a 15 meter RTX-1614 column (0.25 mm ID x 0.10 μm film) was used for the determination of PBDDs. The gas chromatograph was coupled to a Waters Autospec Ultima high-resolution mass spectrometer operated in selected ion monitoring (SIM) mode (positive electron impact, > 10,000 resolution, 32 eV, 280 °C). Acquisition windows were set to include all tetra- through octa-CDD/F isomers.

Windows for di- and tri-CDD/F isomers were centered around the di- and tri-CDD congeners in this study. Therefore, total DCDD and TriCDD values presented should be considered an estimate, as the first and last DCDD and TriCDD eluters may have been outside the established acquisition windows for these isomers. The PBDD analyses included four congeners, 1,3,7-triBDD, 1,2,4,7/1,2,4,8-tetra-BDD and 2,3,7,8-tetra-BDD. Standards for HRGC-HRMS analysis were prepared using a U.S. EPA Method 1613B calibration set (tetra- through octa-CDD/F isomers). A secondary calibration set for di- and tri-CDD/Fs was prepared at similar levels to tetra-CDD/F in the Method 1613B. From these calibration sets, five-point calibration curves were constructed for each PCDD/F congener. Standards for PBDD analysis were prepared at similar levels to tetra-CDD in the Method 1613B using individual native and labeled PBDD standards. A five-point calibration curve was also constructed for each PBDD congener. PBDD results were reported down to th e calibration curve based quantitation limits.
