# Supplementary material for: Quantification of Hydroxylated Polybrominated Diphenyl Ethers (OH-BDEs), Triclosan, and Related Compounds in Freshwater and Coastal Systems
Source: PLoS One. 2015 Oct 14;10(10):e0138805. doi: 10.1371/journal.pone.0138805 (PMC4605494; doi:10.1371/journal.pone.0138805)
Supplement: S2 Appendix — (PDF) [file pone.0138805.s002.pdf]

S2 Appendix

Cleaning Protocols

All glassware and tools were tripled rinsed with diluted Alconox, tap, and deionized (DI) water. Glassware, silica gel, glass wool, sand, and disposable pipettes were baked (550 ^{o}C, 4 hours). Items that would melt at 550 ^{o}C were triple rinsed with ethyl acetate, methanol (MeOH), and acetonitrile (ACN) after the series of water triple rinses. Gas-tight, glass syringes were cleaned by drawing up five aliquots of ethyl acetate, MeOH, and ACN. Each syringe had a designated task – one for each isotope labeled chemical, the 50:50 H_2O:ACN mixture, and transferring extracts. A syringe was cleaned with acetone after each extract transfer.

The solid phase extraction (SPE) sample transfer lines were cleaned with ethyl acetate (20 mL), MeOH (20 mL), and ACN (20 mL). The stainless steel cells for the Accelerated Solvent Extractor (ASE 350; Dionex) underwent a rigorous cleaning procedure. The endcaps were tripled rinsed with tap and DI water, ethyl acetate, MeOH, and ACN. Note that detergents were not used on the endcaps because they degrade the seal. Next, the endcaps were disassembled and sonicated (15 min) in an acetone bath. After being reassembled, they were again triple rinsed with ethyl acetate, MeOH, and ACN. The bodies of the cells underwent the same cleaning as other non-baked glassware.

Equations Used to Calculate Analyte Concentrations in Environmental Samples

The sediment and water concentrations above LOQ were calculated using isotope dilution analysis, an example is shown below for chemical 'X' using peak areas (PA) in

standards (std) and samples. Units for sediments and water were g/L and µM, respectively. First, the response factor (RF) was calculated using:

$$RF = \frac{1}{[{}^{13}C_{12}X_{std}] \cdot m}$$

where ${}^{13}C_{12}X_{std}$ is the concentration of isotope labeled compound in standards and m is the calibration slope described with the following equation:

$$m = \frac{X_{sample}^{PA}}{{}^{13}C_{12}X_{std}^{PA} \cdot [X_{std}]}$$

The mass of X in sediments or concentration in sediments were calculated using:

$$X_{sample}(g\ or\ \mu M) = RF \times \frac{X_{sample}^{PA}}{{}^{13}C_{12}X_{sample}^{PA}} \times {}^{13}C_{12}X_{sample}(g\ or\ \mu M)$$

where ${}^{13}C_{12}X_{sample}$ is the amount of isotope labeled chemical spiked in the sample matrix. The relative recovery for each compound was calculated using the following equation:

$$RelativeRecovery = \frac{X_{spiked,sample} - X_{sample,avg}}{X_{spiked}} \times 100$$

The sediment concentration was calculated using the normalized amount of sediment analyzed in the extract. Sediment and water concentrations were relative recovery corrected. The internal standard for triclosan and 6-OH-BDE 47 and 6’-OH-BDE 100 was its isotope labeled counterpart. ${}^{13}C_{12}$-6’-OH-BDE 100 was the internal standard for all of the OH-PentaBDEs. The absolute recovery (AbsRec) of internal standards was calculated using:

$$AbsRec = \frac{{}^{13}C_{12}X_{sample}^{PA}}{{}^{13}C_{12}X_{std}^{PA}(avg)} \times \frac{[{}^{13}C_{12}X_{std}]}{[{}^{13}C_{12}X_{sample}]} \times 100$$

where $^{13}C_{12}X^{PA}_{std}$ (avg) is the average peak area of isotope labeled compound in standards.
