# Supplementary material for: Quantification of Hydroxylated Polybrominated Diphenyl Ethers (OH-BDEs), Triclosan, and Related Compounds in Freshwater and Coastal Systems
Source: PLoS One. 2015 Oct 14;10(10):e0138805. doi: 10.1371/journal.pone.0138805 (PMC4605494; doi:10.1371/journal.pone.0138805)
Supplement: S1 Fig — (PDF) [file pone.0138805.s003.pdf]

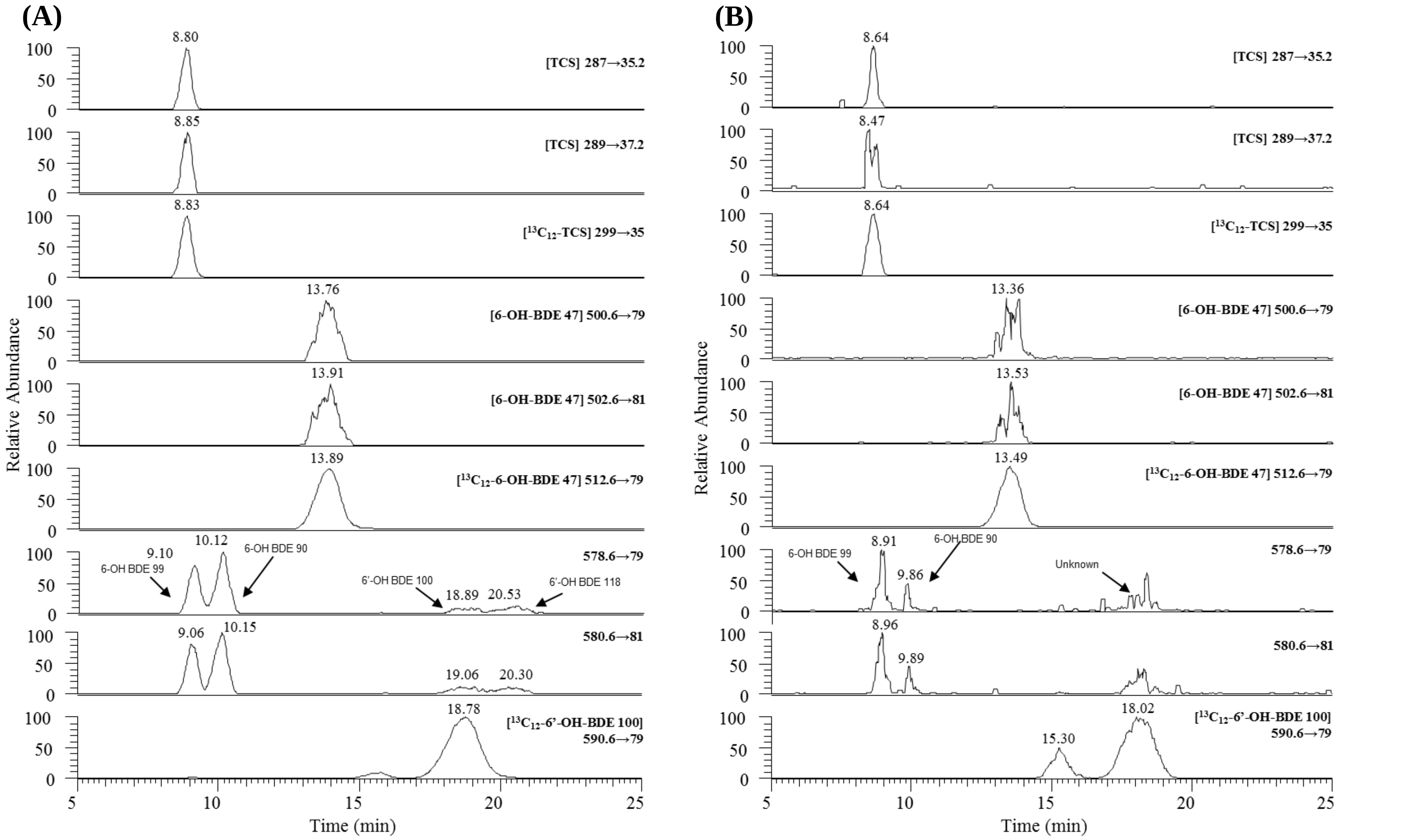

S1 Figure. Representative chromatograms using LC-MS/MS method displaying SRM transitions and retention times for: (A) a standard; and (B) Central Bay 38 – 40 cm sediment. Note that in (B) the peak at 18.02 min in 578.6 → 79 and 580.6 → 81 frames is an unknown and not 6'-OH-BDE 100, because this sample was pre-BDE production.
