# Supplementary figures and images for: Quantification of Hydroxylated Polybrominated Diphenyl Ethers (OH-BDEs), Triclosan, and Related Compounds in Freshwater and Coastal Systems
Source: PLoS One. 2015 Oct 14;10(10):e0138805. doi: 10.1371/journal.pone.0138805 (PMC4605494; doi:10.1371/journal.pone.0138805)

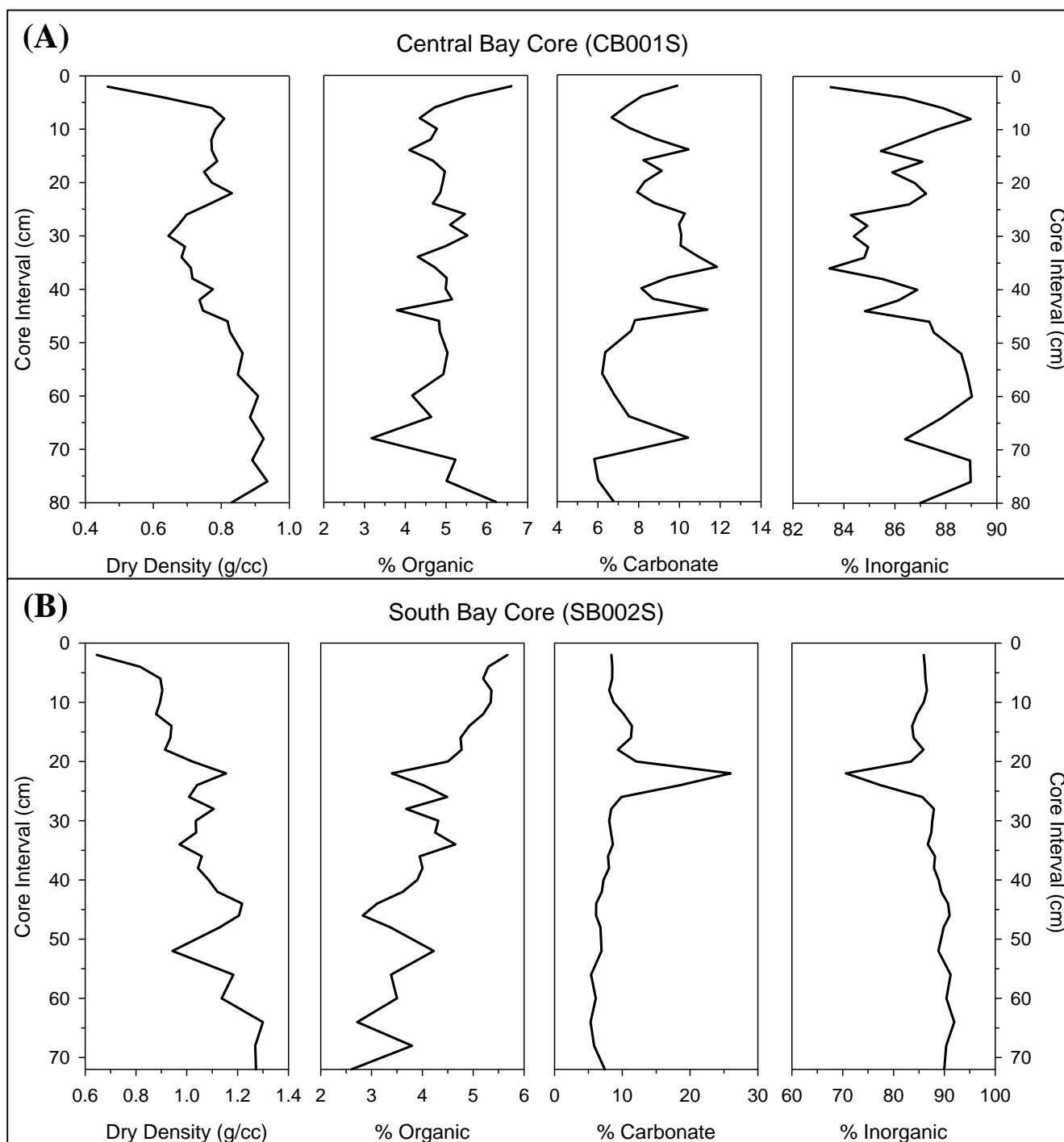

**S2 Figure. Loss-on-ignition results for: (A) Central Bay (CB001S) core, and (B) South Bay (SB002S) core.**

Supplement: S2 Fig — (PDF) [file pone.0138805.s004.pdf]
