# Supplementary material for: Quantification of Hydroxylated Polybrominated Diphenyl Ethers (OH-BDEs), Triclosan, and Related Compounds in Freshwater and Coastal Systems
Source: PLoS One. 2015 Oct 14;10(10):e0138805. doi: 10.1371/journal.pone.0138805 (PMC4605494; doi:10.1371/journal.pone.0138805)
Supplement: S3 Fig — (PDF) [file pone.0138805.s005.pdf]

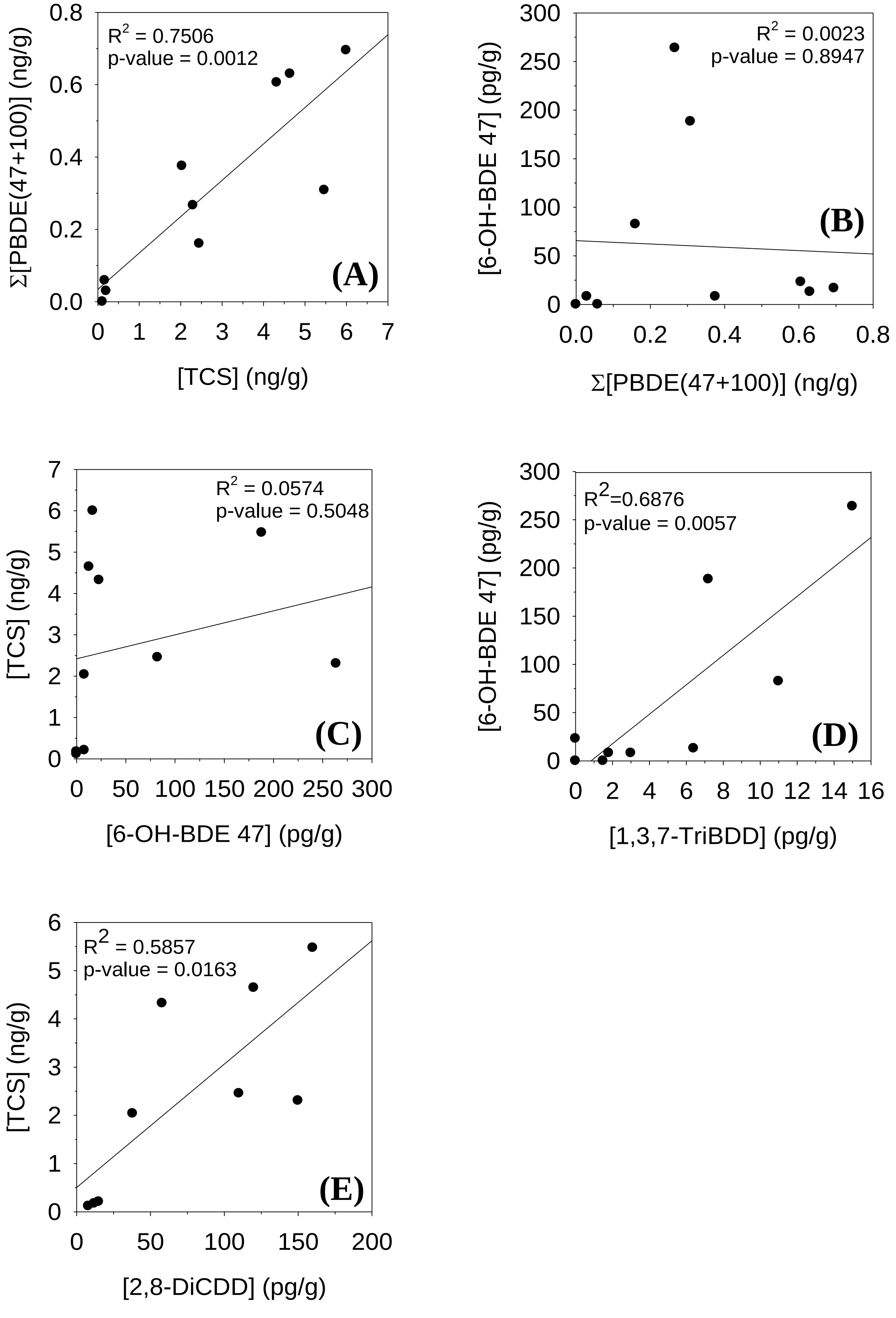

S3 Figure. Graphs displaying a: (A) significant correlation between ∑PBDE(47+100) and triclosan (TCS); (B) insignificant correlation between 6-OH-BDE 47 and ∑PBDE(47+100); (C) insignificant correlation between triclosan and 6-OH-BDE 47; (D) significant correlation between 6-OH-BDE 47 and 1,3,7-TriBDD; and (E) significant correlation between triclosan and its dioxin (2,8-DiCDD) in San Francisco Bay surface sediments.
