# Supplementary material for: Quantification of Hydroxylated Polybrominated Diphenyl Ethers (OH-BDEs), Triclosan, and Related Compounds in Freshwater and Coastal Systems
Source: PLoS One. 2015 Oct 14;10(10):e0138805. doi: 10.1371/journal.pone.0138805 (PMC4605494; doi:10.1371/journal.pone.0138805)
Supplement: S4 Fig — (PDF) [file pone.0138805.s006.pdf]

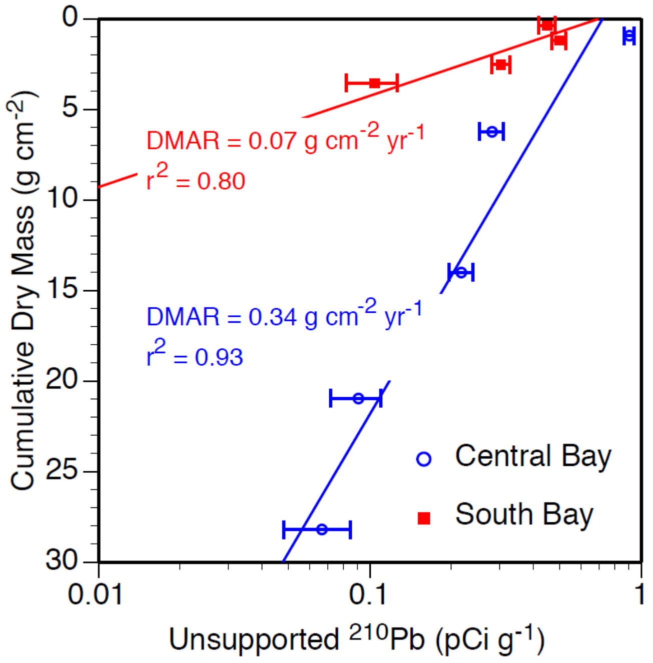

S4 Figure. An approximate chronology was determined for the San Francisco Bay cores by assuming a constant sediment flux (DMAR; dry mass accumulation rate) fitted to the data by least-squares regression (the cf:cs model).
