# Supplementary material for: Quantification of Hydroxylated Polybrominated Diphenyl Ethers (OH-BDEs), Triclosan, and Related Compounds in Freshwater and Coastal Systems
Source: PLoS One. 2015 Oct 14;10(10):e0138805. doi: 10.1371/journal.pone.0138805 (PMC4605494; doi:10.1371/journal.pone.0138805)
Supplement: S1 Table — (PDF) [file pone.0138805.s007.pdf]

S1 Table. Selected reaction monitoring transitions (SRM) for chemical quantification (Q) and confirmation (C).

**Analyte**	**SRM (m/z)**	**Purpose**
TCS	287 → 35.2	Q
	289 → 37.2	C
6-OH-BDE 47	500.6 → 79	Q
	502.6 → 81	C
OH-PentaBDEs	578.6 → 79	Q
	580.6 → 81	C
$^{13}C_{12}$-TCS	299 → 35.2	Q
$^{13}C_{12}$-6-OH-BDE 47	512.6 → 79	Q
$^{13}C_{12}$-6’-OH-BDE 100	590.6 → 79	Q
