# Supplementary material for: Quantification of Hydroxylated Polybrominated Diphenyl Ethers (OH-BDEs), Triclosan, and Related Compounds in Freshwater and Coastal Systems
Source: PLoS One. 2015 Oct 14;10(10):e0138805. doi: 10.1371/journal.pone.0138805 (PMC4605494; doi:10.1371/journal.pone.0138805)
Supplement: S2 Table — (PDF) [file pone.0138805.s008.pdf]

S2 Table. Absolute and relative recovery for $^{13}C_{12}$-PXDDs and PXDDs, respectively, in sediments.

PXDD Recovery

	Absolute Recovery (%)		
**Sediment**	**$^{13}C_{12}$-2,3,7,8-TeBDD**	**$^{13}C_{12}$-2,8-DiCDD**	**n [a]**
San Francisco Bay Surface Sediments	72 ± 13	55 ± 7	8
Point Reyes National Seashore	80 ± 15	43 ± 7	9
**Analyte**	**Relative Recovery (%)**	**%RPD [b]**	**n**
1,3,7-TriBDD	79 - 82	3.7	2
1,2,4,7/1,2,4,8-TeBDD	95 - 99	4.1	2
2,3,7,8-TeBDD	104 - 106	1.9	2
2,8-DiCDD	113 – 120	6	2

[a] n = number of samples

[b] RPD = relative percent difference between matrix spike relicates
