# Supplementary material for: Quantification of Hydroxylated Polybrominated Diphenyl Ethers (OH-BDEs), Triclosan, and Related Compounds in Freshwater and Coastal Systems
Source: PLoS One. 2015 Oct 14;10(10):e0138805. doi: 10.1371/journal.pone.0138805 (PMC4605494; doi:10.1371/journal.pone.0138805)
Supplement: S3 Table — (PDF) [file pone.0138805.s009.pdf]

S3 Table. Loss-on-ignition results for San Francisco Bay surface sediments and Point Reyes National Seashore cores.

Sample ID	Top Depth (cm)	Bottom Depth (cm)	Dry Density (g/cc)	% Organic	% Carbonate	% Inorganic
BG20	0	5	1.29	1.32	1.95	96.7
BG30	0	5	0.85	5.76	4.23	90.0
SU044S	0	5	1.05	2.54	3.33	94.1
SPB001S	0	5	0.61	5.99	6.84	87.2
CB001S	0	5	0.42	6.71	7.94	85.4
SB023S	0	5	0.77	4.69	42.63	52.7
SB002S	0	5	0.70	5.08	7.33	87.6
LSB001S	0	5	N/A	N/A	N/A	N/A
LSB042S	0	5	0.54	5.49	7.88	86.6
BA10	0	5	0.76	4.32	12.32	83.4
Point Reyes A	0	5	1.43	1.17	1.93	96.9
	5	10	1.39	1.67	2.15	96.2
	10	15	1.46	1.56	2.01	96.4
Point Reyes B	0	5	1.42	1.53	1.91	96.6
	5	10	1.15	2.66	2.75	94.6
	10	15	1.53	1.35	1.73	96.9
Point Reyes C	0	5	1.38	1.20	1.96	96.8
	5	10	1.25	1.92	2.22	95.9
	10	16	1.28	1.95	2.31	95.7
