# Supplementary material for: Quantification of Hydroxylated Polybrominated Diphenyl Ethers (OH-BDEs), Triclosan, and Related Compounds in Freshwater and Coastal Systems
Source: PLoS One. 2015 Oct 14;10(10):e0138805. doi: 10.1371/journal.pone.0138805 (PMC4605494; doi:10.1371/journal.pone.0138805)
Supplement: S4 Table — (PDF) [file pone.0138805.s010.pdf]

S4 Table. **Latitude and longitude of salinity measurements for surface water sampling sites near the sediment sampling sites.**

Water				Sediment
Site Name	Sample ID	Latitude	Longitude	Sample ID
Suisun Bay	SU041W	38.09582	-122.0630	SU044S
San Pablo Bay	SPB033W	38.08548	-122.38517	SPB001S
Central Bay	CB033W	37.87172	-122.36898	CB001S
South Bay	SB063W	37.69370	-122.22285	SB023S
South Bay	SB062W	37.54092	-122.16820	SB002S
Lower South Bay	LSB053W	37.49253	-122.09265	LSB001S
Lower South Bay	LSB052W	37.47805	-122.09085	LSB042S
Lower South Bay	LSB054W	37.46833	-122.06432	BA10
